# Intrinsic Thermal Sensing Controls Proteolysis of *Yersinia* Virulence Regulator RovA

**DOI:** 10.1371/journal.ppat.1000435

**Published:** 2009-05-15

**Authors:** Katharina Herbst, Matthias Bujara, Ann Kathrin Heroven, Wiebke Opitz, Martin Weichert, Ariane Zimmermann, Petra Dersch

**Affiliations:** 1 Institut für Mikrobiologie, Technische Universität Braunschweig, Braunschweig, Germany; 2 Abteilung Molekulare Infektionsbiologie, Helmholtz-Zentrum für Infektionsforschung, Braunschweig, Germany; Tufts University School of Medicine, United States of America

## Abstract

Pathogens, which alternate between environmental reservoirs and a mammalian host, frequently use thermal sensing devices to adjust virulence gene expression. Here, we identify the *Yersinia* virulence regulator RovA as a protein thermometer. Thermal shifts encountered upon host entry lead to a reversible conformational change of the autoactivator, which reduces its DNA-binding functions and renders it more susceptible for proteolysis. Cooperative binding of RovA to its target promoters is significantly reduced at 37°C, indicating that temperature control of *rovA* transcription is primarily based on the autoregulatory loop. Thermally induced reduction of DNA-binding is accompanied by an enhanced degradation of RovA, primarily by the Lon protease. This process is also subject to growth phase control. Studies with modified/chimeric RovA proteins indicate that amino acid residues in the vicinity of the central DNA-binding domain are important for proteolytic susceptibility. Our results establish RovA as an intrinsic temperature-sensing protein in which thermally induced conformational changes interfere with DNA-binding capacity, and secondarily render RovA susceptible to proteolytic degradation.

## Author Summary

Temperature is one of the most crucial environmental signals sensed by pathogens to adjust expression of their virulence factors and host survival programs after entry from a cold external environment into a warm-blooded host. Thermo-induced structural changes in bent or supercoiled DNA or mRNA secondary structures are frequently used to modulate virulence gene transcription or translation. Here we introduce a unique alternative mechanism, in which a central regulator of *Yersinia* virulence (RovA) uses an in-built thermosensor to control its activity in order to modulate virulence gene expression. According to our results, small thermo-induced structural alterations reduce the DNA-binding capacity of the virulence regulator and render the protein more susceptible to proteolytic degradation by ATP-dependent proteases. Amino acids in the vicinity of the DNA-binding region appear to comprise the information required for proteolysis. We therefore postulate a model in which proteolytic degradation is in direct competition with the thermo-sensitive DNA-binding function of the regulator. This regulatory concept constitutes a new example of how microbial pathogens are able to rapidly adjust virulence-associated processes in the course of an infection.

## Introduction

Most microbial pathogens occupy different ecological niches inside and outside their mammalian hosts. The temperature shift during entry from the surrounding biosphere or vector reservoirs, where temperature is generally lower than 30°C, into a thermally controlled host environment of 37°C is an important signal informing microbial pathogens to adjust their virulence programs [1]. Most pathogenic bacteria, including the yersiniae, have evolved sophisticated systems to sense the temperature of their environment [2],[3].

*Yersinia pestis* is the etiological agent of plague and leads a sheltered lifestyle, alternately growing in fleas at moderate temperatures or in mammalian hosts at 37°C [4]. *Yersinia pseudotuberculosis* and *Yersinia enterocolitica* are fecal-oral pathogens that survive in moist environments. They are transmitted via ingestion, and cause gut-associated diseases, including enteritis, mesenteric lymphadenitis and diarrhoea [5],[6]. Past studies addressing thermo-dependent changes in pathogenic yersiniae revealed that shifts between moderate temperatures and 37°C result in a global transition of gene expression, including multiple metabolic and stress adaptation genes, and most *Yersinia* virulence factors [7],[8]. Among the virulence properties that are strongly expressed at environmental temperatures but weakly at 37°C are the heat-stable *Y. enterocolitica* specific enterotoxin Yst, iron-scavenging systems, smooth lipopolysaccharides, and the production of the primary internalization factor invasin in the enteropathogenic *Yersinia* species [3],[7]. These characteristics seem to support initial colonization, penetration and survival in host tissues that are encountered during the very early stages of infection. While these properties are repressed at 37°C, expression of the virulence plasmid-encoded type III secretion system, the antiphagocytic Yop proteins and the adhesin YadA are induced [9],[10]. This ensures that the bacteria remain cell adherent, become serum-resistant and are prepared for contact with phagocytic cells of the host immune system during ongoing infections. Although most *Yersinia* pathogenicity factors are thermally regulated, the devices for sensing temperature alterations and the molecular mechanisms how this sensory event is transmitted to globally regulate pathogenicity-associated pathways are not fully understood. Hitherto, it has only been shown that the virulence modulator YmoA of *Y. pestis*, is rapidly degraded by the Lon and ClpP proteases at 37°C, but not at 25°C. Furthermore, a thermo-responsive hairpin structure (“thermoswitch”) has been postulated within the transcript of the transcriptional regulator VirF. Both, YmoA and VirF, control the expression of the type III secretion machinery, translocating the antiphagocytic Yop effector proteins [11],[12].

In this report, we analyze the thermoregulation of the global *Yersinia* virulence regulator RovA. The RovA protein belongs to the SlyA/Hor/Rap family of dimeric winged-helix DNA-binding proteins, which control a wide range of physiological processes implicated in environmental adaptation, survival, and pathogenesis in humans, animals and plants [13],[14]. In pathogenic yersiniae, RovA coordinates the expression of multiple metabolic, stress and virulence genes, which contribute to colonization, host-associated stress adaptation and persistence [15]. In both enteropathogenic *Yersinia* species, RovA activates the transcription of the internalization factor invasin and other adhesins, allows a more efficient colonization of the Peyer's patches, and leads to a faster progression of the infection [16]–[18]. Moreover, dissemination of a *Y. pestis rovA* mutant to liver and lungs was reported to be drastically reduced, and its LD_50_ was shown to be about 80-fold higher than that of the wild-type, demonstrating that the RovA regulator protein is also important for the development of the bubonic plague [19].

The *rovA* gene is transcribed by two promoters in *Y. pseudotuberculosis* and *Y. pestis*, and by three promoters in *Y. enterocolitica*, at moderate temperatures (20–25°C), but is not expressed at 37°C [20],[21]. Transcription of *rovA* is also modulated by growth phase and composition of the culture medium, and is positively autoregulated. Multiple RovA molecules bind to an extended AT-rich sequence far upstream in the *rovA* regulatory region and this interaction is required for full activation of *rovA* transcription [20].

Recent studies showed that *rovA* expression is subject to silencing by the nucleoid-associated protein H-NS [20],[22],[23]. Transcription of the *rovA* gene is also repressed by the LysR-type regulator RovM, which itself is controlled by the carbon storage regulator system of *Yersinia*, implicating small regulatory RNAs [18],[24]. However, none of these established mechanisms can explain the thermal regulation of RovA synthesis. Here we report, that RovA represents a novel proteinaceous thermometer that senses temperature shifts directly through alterations in protein conformation thereby modulating its DNA-binding capacity. We further show that RovA is also subject to temperature and growth phase-dependent degradation by the self-compartmentalized proteases Lon and ClpP.

## Results

### Post-transcriptional control of *rovA* expression

**Figure 1:**
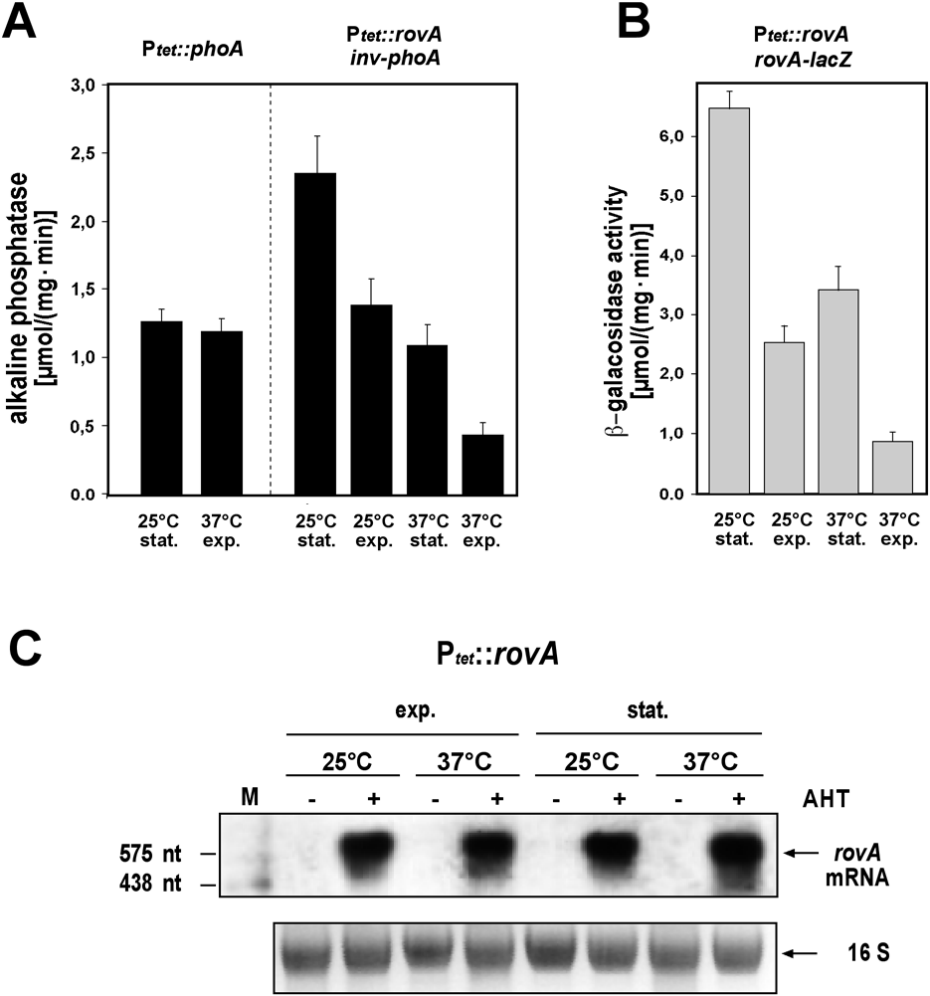
Temperature and growth phase-dependent *rovA* expression occurs on the post-transcriptional level. (A, left panel) Analysis of the expression of the *phoA* gene under the control of the tetracycline promoter (P*_tet_*). Quantification of the expression of the *inv-phoA* fusion (A, right panel), and the *rovA-lacZ* fusion (B) in *E. coli* strain DH5αZ1 pHT123, in which *rovA* is being expressed under the control of the tetracycline promoter (P*_tet_*). Strain DH5αZ1, encoding the *tet* repressor gene *tetR*, was grown overnight or to exponential phase at 25°C or 37°C in the presence of the inducer AHT (0,1 µg ml^−1^). β-galactosidase and alkaline phosphatase activity is the mean of at least three independent determinations done in triplicate ±SD. (C) DH5αZ1 pHT123 was grown in the presence (+) or absence (−) of the inducer AHT 0,1 µg ml^−1^ under indicated growth conditions. Total RNA was prepared, separated on a 0.7% agarose gel, transferred onto a Nylon membrane and probed with a digoxigenin (DIG)-labelled PCR fragment encoding the *rovA* gene. 16 rRNA was used as a loading control. A DIG-labelled RNA marker is loaded in the left lane (M), and arrows indicate the *rovA* mRNA transcript.

Although multiple regulatory factors were shown to be implicated in the environmental control of *rovA* transcription [18],[20],[24], expression studies, in which *rovA* was expressed from the tetracycline promoter (P*_tet_*) in *E. coli*, indicated that temperature and growth phase control of *rovA* expression occurs predominantly on the post-transcriptional level (Figure **1A,B**). RovA-dependent *inv-phoA* and *rovA-lacZ* fusions were still repressed under *rovA* non-inducing conditions (37°C, exponential phase), even when *rovA* was transcribed from P*_tet_*. However, P*_tet_* driven expression of the *phoA* gene showed that P*_tet_* per se was not induced under these environmental conditions. We also performed Northern blot analysis and found identical amounts of the *rovA* mRNA expressed from P*_tet_* at 25°C or 37°C during both growth phases (Figure **1C**). This argued that the amount of RovA in the bacterial cell is a function of temperature and growth phase, which does not depend on regulation of transcription.

### RovA-mediated DNA binding is temperature-dependent

**Figure 2:**
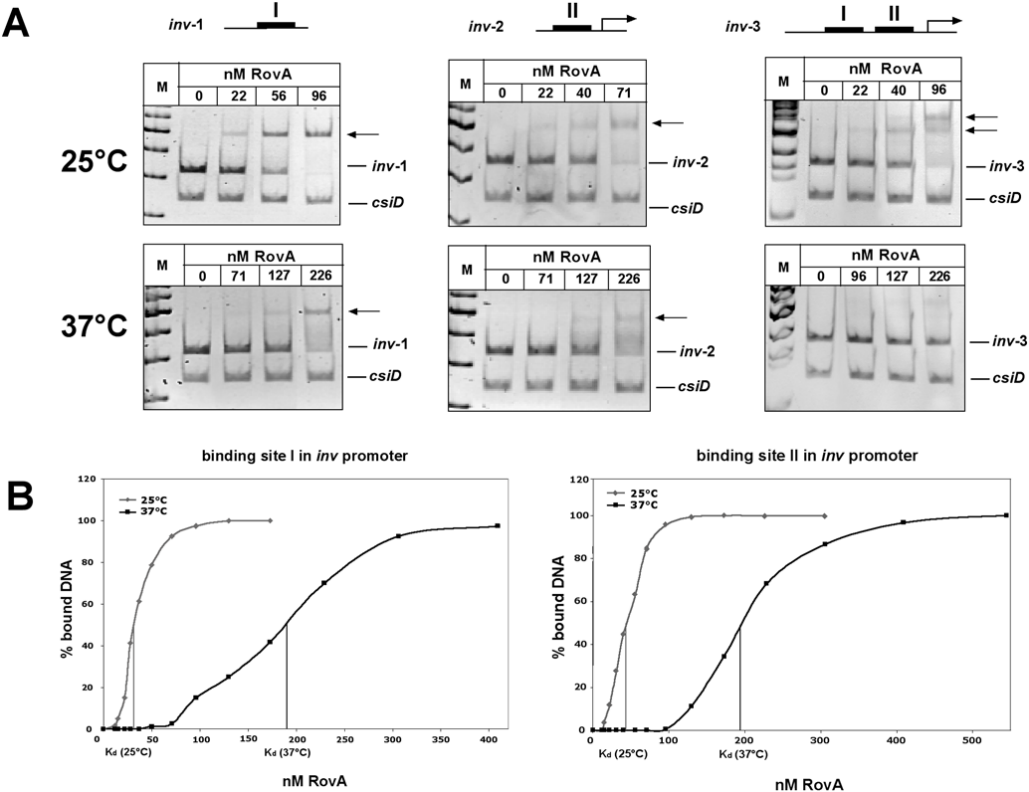
Interaction of RovA with the *inv* regulatory region at 25°C or 37°C. (A) The double-stranded promoter fragments of the *inv* regulatory region harbouring all RovA binding sites, or only the binding site(s) in region I or II (black boxes) were incubated without or with rising amounts of purified RovA protein at 25°C or 37°C. The DNA-RovA complexes were separated on a 4% polyacrylamide gel. A non-specific probe containing an unrelated sequence (*csiD* promoter of *E. coli*) was included as negative control. Different DNA fragments used for the band shift assays are illustrated on top. Bold lines indicate previously identified RovA binding sites. Band shift analyses of the chosen fragments are shown below. (B) A constant concentration of DNA fragments, harbouring site I or II of the *inv* promoter region, were incubated with different increasing concentrations of RovA and subjected to band shift assays. The DNA bands were quantified and the K_d_ was defined as the protein concentration required for half-maximal binding and was calculated for each protein-DNA concentration and presented as average of at least four independent experiments.

**Figure 3:**
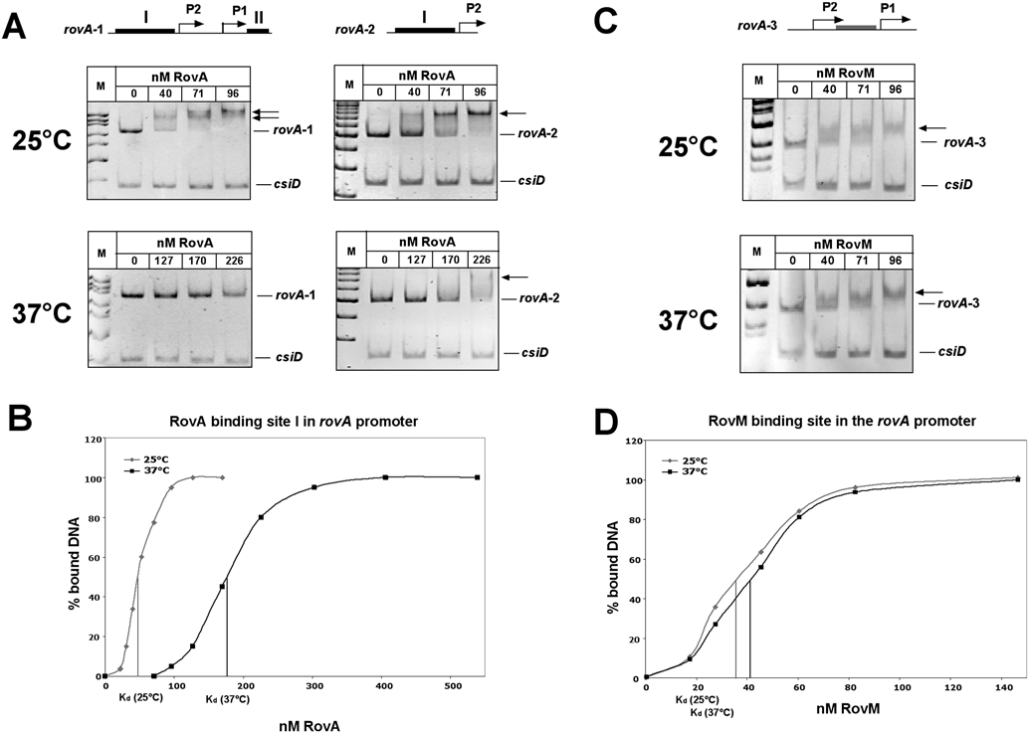
Interaction of RovA or RovM with the *rovA* regulatory region at 25°C or 37°C. Double-stranded promoter fragments of the *rovA* regulatory region harbouring RovA binding sites (black boxes) or the RovM binding site (grey box) were incubated without or with rising amounts of purified RovA protein (A) or RovM (C) at 25°C or 37°C. The DNA-protein complexes were separated on a 4% polyacrylamide gel. A non-specific probe containing an unrelated sequence (*csiD* promoter of *E. coli*) was included as negative control. Different DNA fragments used for the band shift assays are illustrated on top. Bold lines indicate previously identified RovA or RovM binding sites. Band shift analyses of the chosen fragments are shown below. (B, D) A constant concentration of DNA fragments, harbouring RovA binding site I or the RovM binding site of the *rovA* promoter region, were incubated with different increasing concentrations of RovA (B), or RovM (D) and subjected to band shift assays. The DNA bands were quantified and the K_d_ was defined as the protein concentration required for half-maximal binding and was calculated for each protein-DNA concentration and presented as average of at least four independent experiments.

Genetic screens to identify components that up- or down-regulate *rovA* expression in response to temperature or attempts to show temperature-dependent modification of RovA, e.g. by 2D gel electrophoresis and phosphospecific fluorescent dyes failed. We therefore hypothesized that RovA might act as a protein thermometer, which is able to sense temperature of its environment directly through alteration in protein conformation without involvement of other cellular components. To test this, we first performed RovA DNA-binding experiments at 25°C and 37°C with different *inv* or *rovA* promoter fragments, harbouring one or more RovA binding sites. To demonstrate specificity of RovA binding, a control fragment encoding the *csiD* gene of *E. coli* was also introduced into the assay. The DNA-binding behaviour of RovA is significantly different at both temperatures, in such that the RovA protein binds DNA *in vitro* more avidly at low temperature. As illustrated in Figure **2A**, significantly more of the same protein sample was required to cause the disappearance of free *inv* promoter fragments and the appearance of higher molecular RovA-DNA complexes at 37°C. To quantify these effects, we carried out band shift assays with a wide range of RovA concentrations, which allowed us to determine the apparent dissociation constant (K_d_). We found that at 25°C the RovA protein interacts with a K_d_ of about 32±5 nM and 45±4 nM with the binding sites I and II of the *inv* promoter, but it exhibits a considerably lower affinity (K_d_ of about 183±24 nM and 190±23 nM) to the same binding sites at 37°C (Figure **2B**). A similar thermo-induced reduction of RovA DNA binding was seen with *rovA* promoter fragments, harbouring all or only the RovA binding region I upstream of promoter P2 (Figure **3A**). RovA affinity to binding site I was about 4-fold reduced upon a temperature shift and occurred with a K_d_ of 46±3 nM at 25°C and a K_d_ of 178±5 nM at 37°C (Figure **3B**). Interestingly, the defect in RovA binding was especially apparent, when multiple RovA binding sites were present. No or only a very small amount of the highest molecular weight RovA-DNA complexes could be detected with the *inv* and *rovA* fragments, containing two or more RovA binding sites, implying that particularly cooperative binding of RovA on DNA is strongly reduced at 37°C.

To further demonstrate that this temperature-dependent difference is a RovA specific property, we investigated DNA-binding properties of the RovM protein, which also interacts with the *rovA* promoter region. In contrast to RovA, no significant difference in the DNA-binding activity to the *rovA* promoter fragment was observed (Figure **3C**). At all tested RovM concentrations, RovM DNA-binding at 25°C (K_d_ of 36±6 nM) was similar or only slightly lower compared with 37°C (K_d_ of 41±3 nM) (Figure **3D**). A pH shift from 7.3 to 7.8, covering the small temperature-induced increase in the band shift buffer system also had no effect on RovA DNA-binding at 25°C (data not shown). This strongly indicated that observed thermo-dependent changes of RovA DNA-binding are not simply the result of thermo-dynamic effects or thermo-related changes in the pH. We also analyzed the DNA-binding capability of RovA after it was incubated at 37°C for more than 1 h, and allowed to cool down to 25°C. RovA was not affected by this treatment and exhibited similar DNA-binding properties as the protein which was only incubated at 25°C (data not shown). Hence, incubation at higher temperatures does not irreversibly damage the function of RovA. Furthermore, we found, that loss and gain of function upon temperature up- and downshifts occurred very rapidly (data not shown), suggesting that variations of the temperature between 25°C and 37°C induce conformational changes in RovA, that affect its DNA-binding capacity.

### Temperature-induced conformational changes of RovA

**Figure 4:**
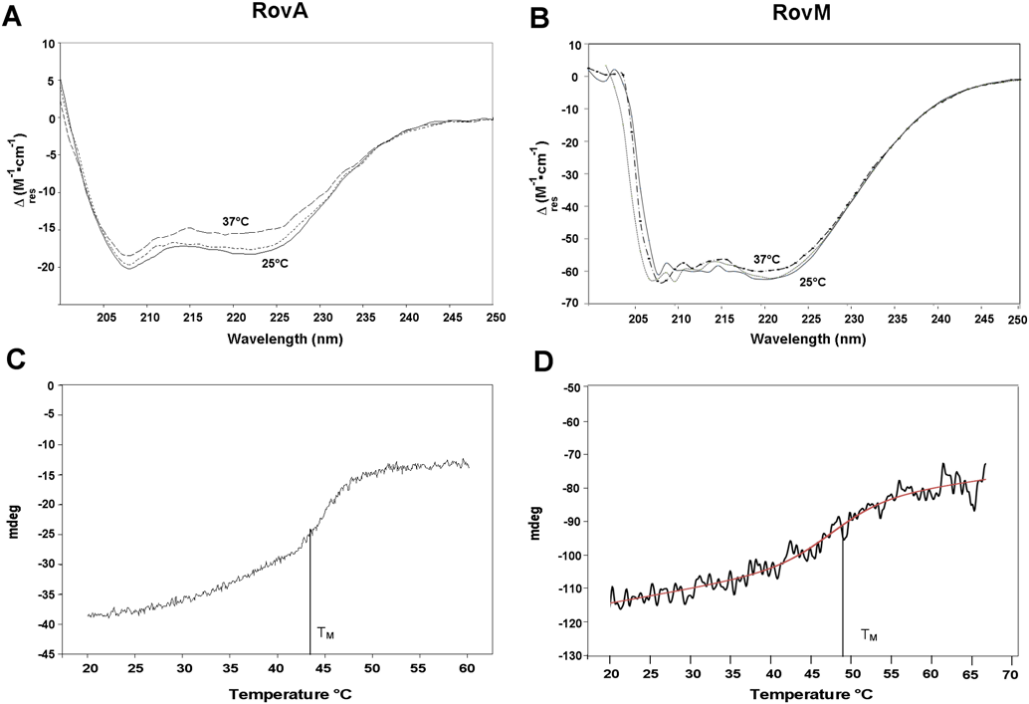
Conformational analysis of RovA and RovM using CD spectroscopy. CS spectra, Δε (M^−1^ cm^−1^) versus wavelength of RovA (0.16 mg/ml) (A) or RovM (0.4 mg/ml) (B) as function of temperature (25°C, solid line; 37°C broken line; 37°C and cooling to 25°C dotted line). Thermal stability of the RovA (C) and the RovM (D) protein. The temperature of the purified protein solutions were increased from 20°C to 60°C with a temperature slope of 2°C/min. The denaturation curves were recorded at fixed wavelength of λ = 222 nm. The melting points (T_M_) were calculated using the Jascow spectra analysis software.

Structural modelling of RovA based on mutant analysis and homologies with other crystallized MarR-type proteins predicted a highly α-helical dimeric protein, in which the first α-helix of one RovA subunit inserts between the last two α-helices of the other [23] (Figure **S1**). Interacting α-helical coiled-coil domains are known to be sensitive to temperature changes [25]. Consequently, loss of RovA DNA-binding functions at 37°C could be the result of thermo-induced conformational changes within the RovA protein. To test this possibility, the temperature effect on the overall conformation of RovA was investigated by using Circular Dicroism (CD) spectroscopy. CD spectroscopy was performed with highly purified RovA between 200nm and 250nm at 25°C and after heating the samples to 37°C (Figure **4A**). The CD spectrum confirmed RovA being a highly α-helical protein (Table **1**). It also showed that a temperature increase to 37°C led to a profound change of the profile, indicating that α-helical structures were lost, whereas the content of β-sheets increased. The CD spectrum stabilized within 10 minutes following the shift to 37°C, indicating that major changes to the structure were complete by this time (data not shown). In contrast, CD spectroscopy of control proteins (RovM Figure **4B**, lysozyme Figure **S2**) did not lead to detectable conformational changes under identical conditions. Furthermore, the overall shape of the CD spectrum of RovA did not change radically, indicating that the overall structure was only partially altered. In fact, reversibility of the thermal unfolding transitions could be demonstrated by the recovery of the initial secondary structure profile. After recooling to 25°C, the spectrum was similar to the original spectrum recorded at 25°C (Table **1**, Figure **4A**).

**Table 1:** Secondary structure of RovA at different temperatures.

Secondary structure	25°C	37°C	25°C after recooling
α-helices	53.5%	41.5%	52.3%
β-sheets	15.5%	25.6%	16.2%
turn	0%	0%	0%
random	31.0%	32.9%	31.5%

Secondary structure components of purified RovA at 25°C, 37°C and after cooling to 25°C were estimated using the Yang algorithm.

Temperature stability of RovA was further investigated using a temperature scan from 20°C to 60°C, and the melting point (T_M_) of RovA was determined to be 44.5°C (Figure **4C**). Stability curves, performed with control proteins, usually proceed without a strong slope to the melting point of the particular protein (Figure **4D**, Figure **S2**). However, a significant gradient between 20°C and 40°C was observed for the RovA regulator protein, also implying conformational changes within this temperature range (Figure **4C**). Taken together, temperature-dependent DNA-binding property seems a consequence of reversible conformational alterations of RovA in the range of 25°C–37°C. Conformational transitions with respect to the surrounding temperature outside and inside hosts could represent a reversible conversion between an active and an inactive form.

### Temperature- and growth phase-dependent stability of RovA

**Figure 5:**
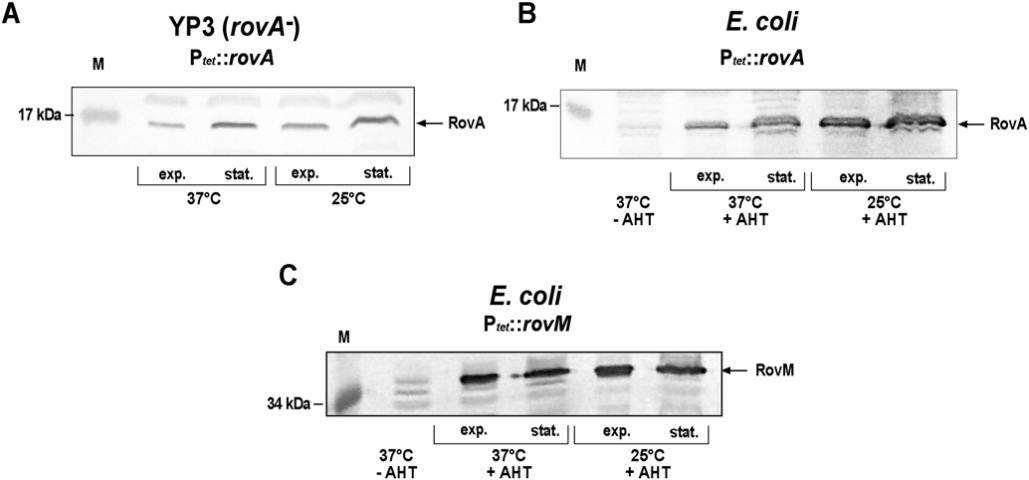
Analysis of the intracellular RovA and RovM levels when expressed under the control of the tetracycline promoter (P*_tet_*). *Y. pseudotuberculosis* strain YP3 pHT123 (A), *E. coli* strain DH5αZ1 pHT123 (B) and *E. coli* strain DH5αZ1 pKH31 (C) were grown overnight or to exponential phase at 25°C or 37°C in the presence of the inducer AHT (0,1 µg ml^−1^). Whole cell extracts from the cultures were prepared, and analyzed by Western blotting with a polyclonal antibody directed against RovA or RovM [20].

**Figure 6:**
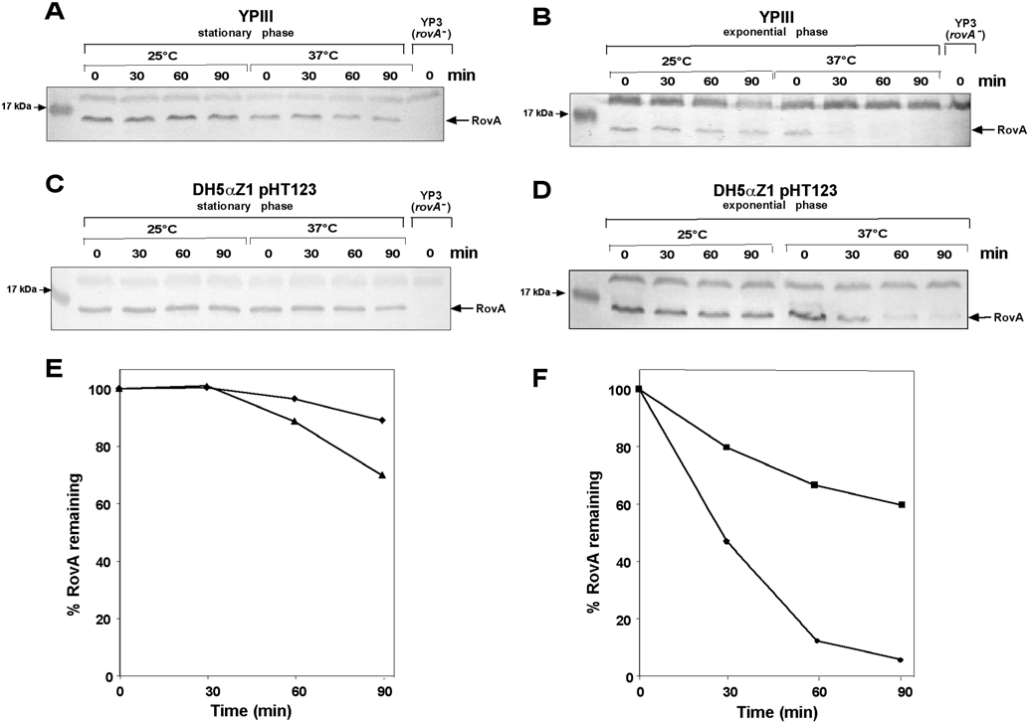
Stability of RovA during exponential and stationary phase at 25°C and 37°C. Cultures of *Y. pseudotuberculosis* strain YPIII (wt) (A–B) and *E. coli* strain DH5αZ1 pHT123 (C–F) were grown overnight or to exponential phase (OD_600_ = 0.3–0.4) at 25°C before chloramphenicol (200 µg ml^−1^) was added. The cultures were divided and incubated at 25°C or 37°C for additional 90 min. Aliquots of the cultures were removed at the indicated times thereafter, whole cell extracts from identical numbers of bacteria were prepared and analyzed by Western blotting with a polyclonal antibody directed against RovA. A whole cell extract from the *rovA* mutant strain YP3 grown overnight at 25°C was used as control. (E, F) Protein bands of DH5αZ1 pHT123 were quantified using the BioRad analysis software ‘Quantity One’ 4.6.2 and set into relation to sampling at time point zero (◆ 25°C, stationary phase; ▲ 37°C, stationary phase; ■ 25°C, exponential phase; ● 37°C, exponential phase).

As the purified RovA protein itself is stable even after several rounds of temperature-induced conformational transitions (Figure **4**), it was expected that similar levels of the RovA protein are produced at 25°C and 37°C when the *rovA* gene is expressed from a foreign promoter to exclude autoactivation. However, lower levels of the RovA protein were detected at 37°C than at 25°C in *Y. pseudotuberculosis* (Figure **5A**) and *E. coli* (Figure **5B**) during exponential and stationary phase, when the *rovA* gene was transcribed from P*_tet_*. In contrast, no alteration of RovM levels was observed, when the *rovM* gene was transcribed from P*_tet_* under the identical growth conditions (Figure **5C**). It is possible that conformational changes upon a temperature shift from 25°C to 37°C lead to an altered stability of the RovA protein *in vivo*. For this reason, we first investigated the influence of different growth temperatures on the stability of RovA in *Y. pseudotuberculosis* YPIII after blockage of the protein biosynthesis. As shown in Figure **6A**, RovA remained stable at 25°C during stationary phase for at least 90 min. At 37°C RovA levels were somewhat reduced, but considerable amounts of RovA were still detectable. During exponential growth, endogenous RovA levels were reduced, as described previously [17]. Furthermore, RovA was rapidly degraded when the culture was shifted to 37°C, and no or only very low amounts of the RovA protein were detectable 30 min after cessation of protein synthesis (Figure **6B**). RovA was significantly more stable at 25°C during log phase as considerable amounts of the regulatory protein were still visible 90 min after protein biosynthesis was stopped. A similar temperature- and growth phase-dependent RovA degradation pattern was also observed with *E. coli* DH5αZ1 pHT123, in which *rovA* was expressed from P*_tet_* to allow a stronger synthesis of the RovA protein during exponential growth (Figure **6C–F**). In contrast, no difference in RovM levels was detected after blockage of protein synthesis in *Y. pseudotuberculosis* and after overexpression of *rovM* from P*_tet_* in *E. coli* under the same conditions (Figure **S3**). These data suggested that RovA is not only inactivated, but is also rapidly degraded under non-inducing conditions, and indicated that a conserved mechanism is responsible for this post-translational control process.

### The ClpP and Lon proteases affect RovA stability

**Figure 7:**
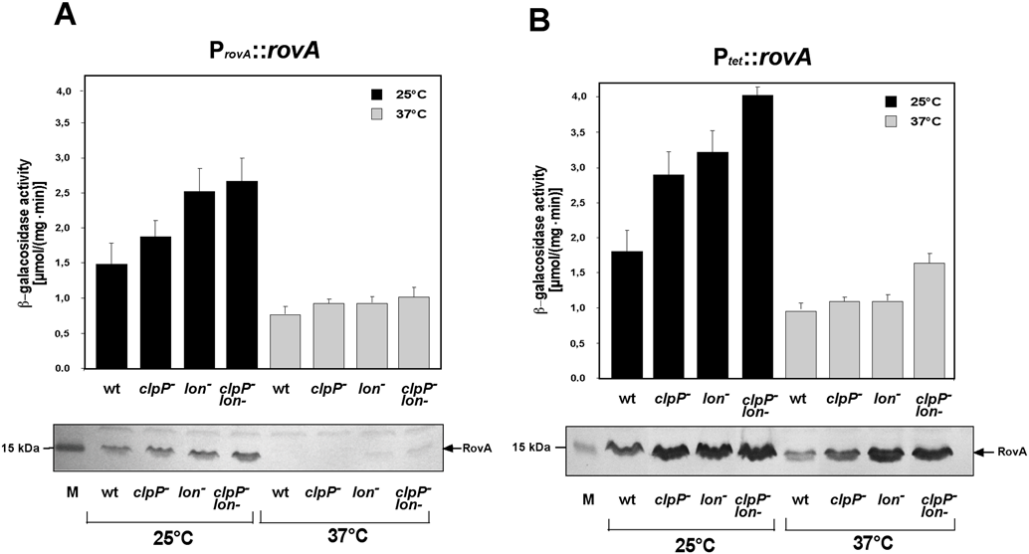
RovA expression in *Y. pseudotuberculosis* wild-type and protease mutants at 25°C and 37°C. Expression of a *rovA-lacZ* fusion on pAKH47 was analyzed in *Y. pseudotuberculosis* YPIII (wt) and in the *clpP*, *lon* and *clpP/lon* mutant strains (YP63, YP67 and YP68), when *rovA* was expressed from its own promoter (P*_rovA_*::*rovA*) (A), and in the presence of the *rovA* expression plasmid pHT123 (P*_tet_*::*rovA*) (B). Cultures were grown at 25°C and 37°C in LB medium overnight and β-galactosidase activity was determined. The data represent the average ±SD from at least three different experiments each done in duplicate (upper panel). Furthermore, whole cell extracts of equal amounts of the bacteria were prepared, separated by SDS-PAGE, and visualized by immunoblotting using a polyclonal antibody directed against RovA.

Pathways implicated in the proteolysis of regulatory components often involve ClpP or Lon proteases. To determine whether these proteases are responsible for RovA degradation, we constructed *Y. pseudotuberculosis clpP*, *lon* and *clpP/lon* deletion mutants and compared expression of a *rovA-lacZ* fusion and steady-state levels of the RovA protein between wild-type and the protease mutants at 25°C and 37°C (Figure **7A**). The *lon* and the *clpP/lon* mutant contained significantly more RovA than the wild-type at 25°C. In contrast, only a slight increase of RovA levels was observed in the *clpP* mutant, suggesting that RovA is mainly degraded by the Lon protease. Interestingly, no or only very low amounts of the RovA protein were found in cultures grown at 37°C, even in the absence of the ClpP and Lon proteases. A similar expression pattern was also observed with a plasmid-based *rovA-lacZ* reporter system. This observation supports our previous results and can be explained by the fact, that the DNA-binding capacity of RovA to its own promoter sequence is strongly reduced at 37°C (Figure **3A**). Thus, autoactivation of *rovA* is abolished at 37°C even in the absence of RovA-degrading proteases.

To confirm our assumption, we repeated this experiment with a construct in which *rovA* was now expressed under the control of P*_tet_* to avoid positive autoregulation (Figure **7B**). In fact, similar levels of the RovA protein were now synthesized at both temperatures, with significantly higher levels in the *lon* and *lon/clpP* mutant, and higher expression of the RovA-dependent *rovA-lacZ* fusion was detected at 25°C in the protease mutants. However, no or very little activation of *rovA* transcription was evident at 37°C, even when RovA is abundant. This implies that not proteolysis, but the effect of thermo-induced conformational changes on DNA-binding is most critical for RovA-dependent regulation of *rovA* expression.

### Lon is mainly required for regulated RovA proteolysis

**Figure 8 ppat-1000435-g008:**
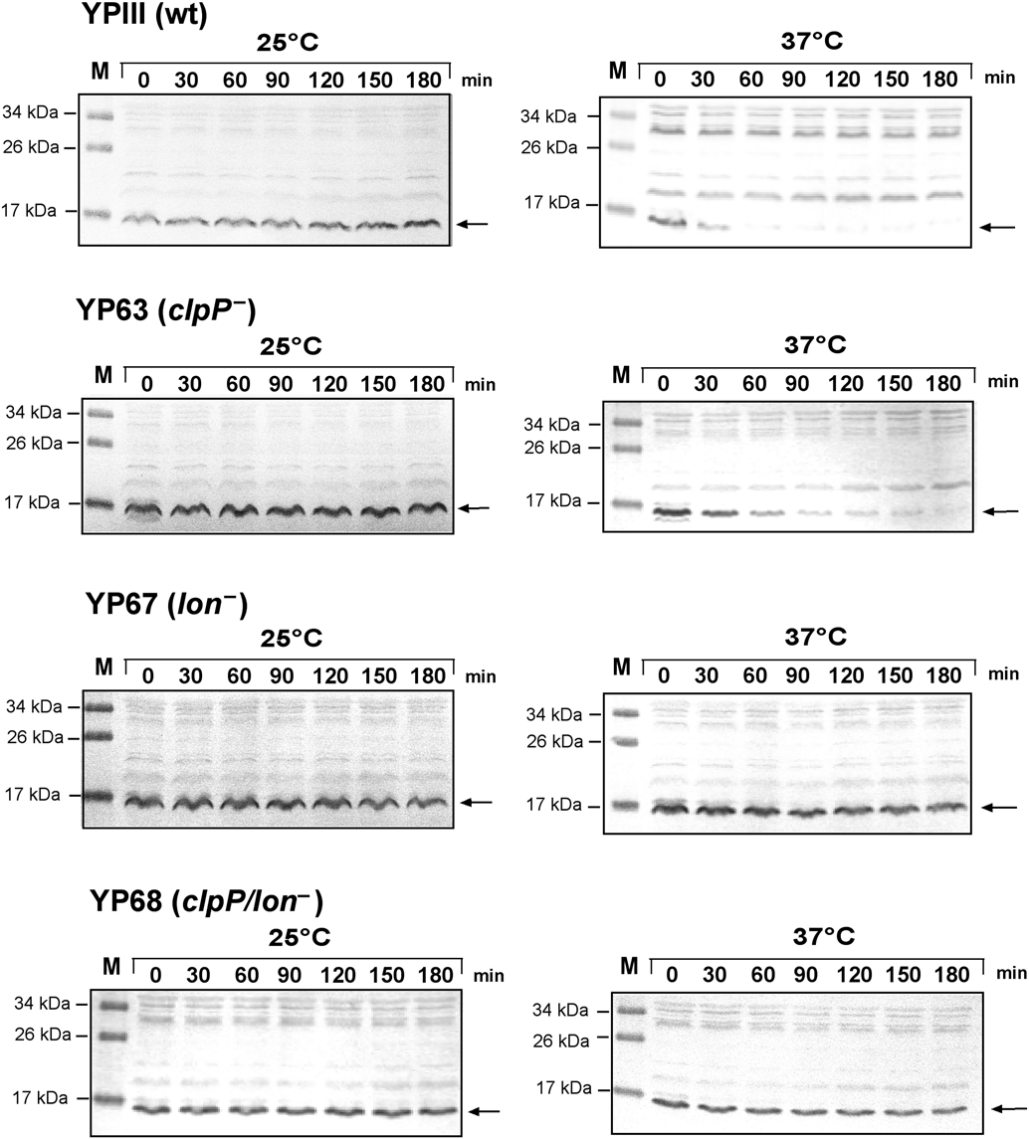
Stability of RovA during exponential phase at 25°C and 37°C in *Y. pseudotuberculosis* YPIII and the *clpP*, *lon*, and *clp/lon* deletion mutants YP63, YP67 and YP68. Cultures of *Y. pseudotuberculosis* strain YPIII (wt) and the *clpP*, *lon*, and *clp/lon* deletion mutants harbouring the P*_tet_*::*rovA* expression plasmid pHT123 were grown to exponential phase (OD_600_ = 0.3–0.5) at 25°C before chloramphenicol (200 µg ml^−1^) was added. The cultures were divided and incubated at 25°C or 37°C for additional 180 min. Aliquots of the cultures were removed at the indicated times thereafter, whole cell extracts from identical number of bacteria were prepared and analyzed by Western blotting with a polyclonal antibody directed against RovA. The arrow indicates the RovA protein.

To test the impact of the ClpP and Lon proteases on RovA stability in more detail, we expressed RovA from P*_tet_* to exclude autoregulation and guarantee efficient RovA production during log phase. RovA stability was first studied in the wild-type and the protease mutants, when incubated at 25°C or 37°C during exponential phase. As shown in Figures **8** and **S4A**, RovA concentrations in the wild-type decreased rapidly at 37°C (t_1/2_ = 30 min), and no or only very low amounts were detectable 1 h after cessation of protein synthesis. Fast degradation of RovA was also observed in the *clpP* mutant at 37°C, although slightly higher RovA levels were detected than in the wild-type background. In contrast, RovA levels were only slightly reduced at 37°C in the *lon* mutant (t_1/2_>3 h), and no degradation of the RovA regulatory protein was detectable in the *clpP/lon* double mutant (Figure **8**, Figure **S4A**). In agreement to previous experiments, no or significantly less degradation was observed in the wild-type and protease mutant cultures which remained at 25°C. This degradation pattern was also observed in bacteria grown to stationary phase, yet at much later time points after block of protein synthesis (Figure **S5**). In summary, albeit ClpP proteases contributed to RovA degradation, proteolysis of RovA was predominantly mediated by the Lon protease.

### An internal portion of the RovA protein is important for regulated proteolysis

**Figure 9 ppat-1000435-g009:**
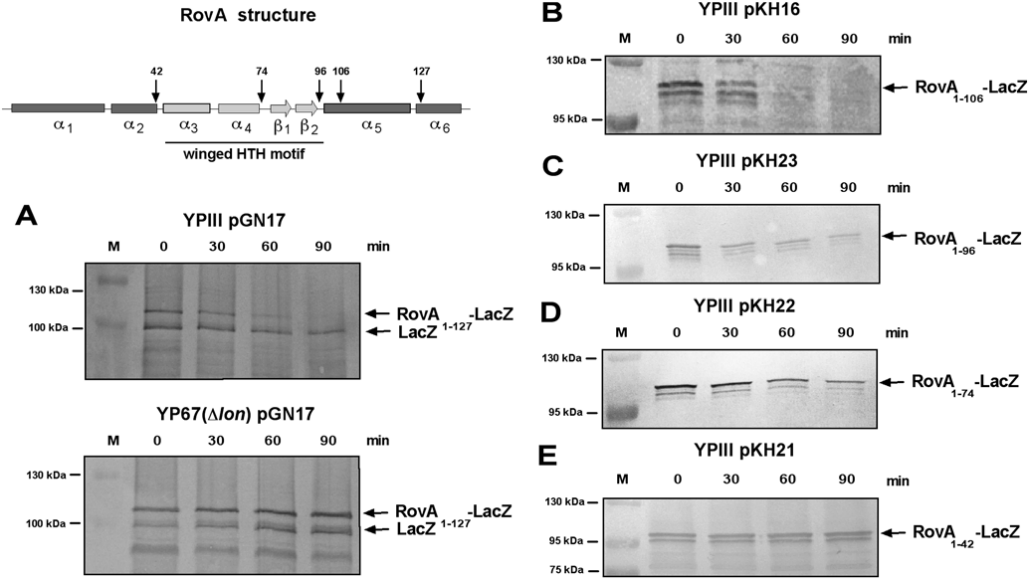
Stability of different RovA-LacZ fusion proteins during exponential phase at 37°C. Cultures of *Y. pseudotuberculosis* strain YPIII (wt) harbouring the *rovA-lacZ* expression plasmid pGN17 (A), pKH16 (B), and pKH21-23 (C–E) and the *lon* deletion mutant YP67 harbouring the *rovA-lacZ* expression plasmid pGN17 (A) were grown to exponential phase (OD_600_ = 0.3–0.4) at 25°C before chloramphenicol (200 µg ml^−1^) was added. The cultures were shifted to 37°C for additional 90 min. Aliquots of the cultures were removed at the indicated times, whole cell extracts from identical numbers of bacteria were prepared and analyzed by Western blotting with a polyclonal antibody directed against β-galactosidase (A) or RovA (B–E). Structure of the RovA protein is given in the upper panel on the left. The predicted α-helical domains implicated in dimerization are given in dark grey, the two α-helices of the HTH motif are indicated by light grey boxes, and the two β-sheets of the winged HTH DNA-binding motif are given in light grey arrows. The numbers above the arrows indicate the last amino acid fused to β-galactosidase.

It has previously been shown that the N- and C-termini of individual proteins can determine the sensitivity to proteolysis. For instance, non-polar amino acids or hydrophobic extensions at the C-termini, e.g. added by the SsrA-tagging system, were shown to destabilize polypeptides [26],[27]. The importance of the N-termini for degradation is represented by the N-end rule, which relates the *in vivo* half-life of a protein to the identity of its N-terminal residues, and was also shown by the instability conferred by an N-terminal fragment of the UmuD protein of *E. coli* or the HemA protein of *Salmonella typhimurium* [28]–[30]. In order to identify amino acid motifs of RovA important for protease susceptibility, we analyzed the stability of terminally His_6_-tagged RovA variants, and tested degradation of different RovA deletion mutants missing the last 5, 23 and 36 C-terminal amino acids [23]. We found that their susceptibility to proteolysis is similar to native RovA (data not shown). We further determined the stability of different RovA-LacZ hybrid proteins. Degradation of the RovA_1–127_-LacZ chimer, harbouring the first 127 amino acids of RovA, was identical to native RovA, and its turnover was blocked in a *lon* mutant (Figures **9A,B** and **S4B**). Continuous deletions of the C-terminal portion of RovA revealed that RovA-LacZ hybrid proteins constituted of the N-terminal 96 or more amino acids were rapidly degraded at 37°C, but not in a *lon* mutant background. A RovA-LacZ fusion protein, including only the first 74 amino acids of RovA, remained more stable at 37°C and was present in considerable amounts even 90 min after blockage of protein synthesis, whereas chimera with the first 42 or less amino acids of RovA were completely stable under the same conditions (Figures **9C–E**, **S4B** data not shown). This demonstrated that addition of the N-terminal 96 residues of RovA to the otherwise stable β-galactosidase is sufficient to signal the chimera for degradation, whereas the first 42 amino acids of the regulatory protein are not. Taken together, this indicated that residues within the center of RovA are important for regulated proteolysis of this protein.

The internal portion of the RovA protein forms the winged-helix DNA-binding domain [23] (Figure **S1**). Previous work in this study showed that DNA-binding is significantly reduced at 37°C, i.e. conditions under which RovA proteolysis is considerably enhanced. This suggested that binding of RovA to DNA might protect the protein from degradation. Accordingly, we determined the effect of multiple copies of the RovA-binding sequences on the stability of native RovA *in vivo*. To do so, we transformed plasmid pKH24 harbouring the sequences including all RovA binding sites of the *rovA* promoter region and the empty vector pUC19 into *Y. psudotuberculosis* YPIII, and determined the stability of the native RovA protein in the exponential phase at 37°C. Presence of multiple copies of the RovA binding sites led to somewhat higher RovA levels (Figure **S6A**). Although RovA was still degraded, low amounts of the regulatory protein were still visible 90 min after blockage of protein synthesis. Furthermore, we found that introduction of an amino acid substitution in the winged helix domain of RovA (RovAE71K), shown to diminish DNA-binding [23], reduced the *in vivo* half-life of the RovA protein during exponential growth at 37°C (Figure **S6A**). This suggested, that RovA is less accessible for the proteases when bound to DNA.

### Degradation of RovA by the Lon protease *in vitro*

**Figure 10:**
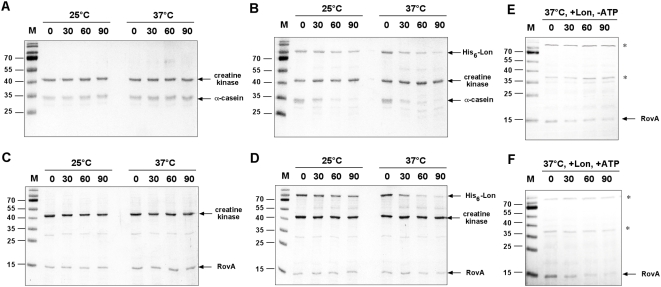
*In vitro* degradation of α-casein and RovA by Lon. α-casein and purified RovA were incubated at 25°C and 37°C in the presence of ATP and an ATP-regeneration system without (A, C) or with (B, D) purified Lon. Whole cell extracts of BL21 (*lon* ^−^) were prepared and added to purified Lon and RovA in the absence (E) and presence of ATP and the ATP regeneration system (F). The degradation reactions were performed as described in Material and Methods. An aliquot was removed at indicated times, separated on 15% SDS-gels, and visualised after staining with Coomassie brilliant blue (A–D) or by Western blotting with an anti-RovA antibody (E–F). * indicates protein bands that interacted non-specifically with the RovA antibody used for loading controls.

The analysis of RovA stability in *Y. pseudotuberculosis* revealed that Lon is the protease primarily responsible for *in vivo* degradation of RovA (Figures **7**–[Fig ppat-1000435-g008] **9**). To establish a direct relationship between RovA degradation and the Lon protease, we tested whether Lon is capable of digesting RovA in an *in vitro* system, containing only purified RovA and Lon of *Y. pseudotuberculosis*, ATP and a system for ATP regeneration. To ensure that purified Lon protease is active, we used purified α-casein as a control substrate, which was known to be also prone to Lon-mediated proteolysis [31]. Figure **10** shows that α-casein was rapidly degraded by the Lon protease at 25°C and 37°C, demonstrating that the purified *Y. pseudotuberculosis* Lon protein is active and able to degrade substrates with similar activities at both temperatures. This indicated that temperature-dependent proteolysis of RovA is not primarily attributed to an increased activity of the Lon protease at 37°C. Quantification of native RovA after *in vitro* degradation further showed that RovA is only very slowly degraded by the Lon protease at 37°C, and remained completely stable at 25°C (Figures **10**, **S4C**). Similar results were also obtained when purified N- or C-terminally His-tagged variants of the *Yersinia* or *E. coli* Lon protein were included in the *in vitro* assay (data not shown).

Slow degradation of RovA in the *in vitro* system suggested that efficient proteolysis *in vivo* requires accessory factors or chaperones, which may affect recognition and enzymatic activity of the Lon protease and/or assist in unfolding of the substrate. To explore whether additional components are required for Lon-mediated proteolysis of RovA, we tested degradation of RovA in a crude extract of exponentially grown *E. coli* strain BL21 (*lon* ^−^) at 37°C after addition of the purified Lon protein of *Y. pseudotuberculosis* in the presence and absence of the ATP regeneration system. Efficient RovA degradation can be observed in the presence of the Lon protease and ATP (Figures **10F**, **S4C**), but not in the absence of ATP or Lon, or with extract from cells grown under conditions that do not result in RovA degradation (Figures **10E**, **S4C** and **S7**). This strongly suggests that one or more conserved accessory factors are essential for RovA proteolysis.

### Synthesis of the Lon protease is temperature-dependent

The *lon* gene in *E. coli* is part of the heat shock regulon and increases after exposure to high temperature (42°C) in a manner that affects overall rates of protein degradation [32],[33]. To test whether also synthesis of the Lon protease is increased in *Y. pseudotuberculosis* under conditions in which RovA is preferentially degraded, we analyzed *lon* expression of *Y. pseudotuberculosis* YPIII at 25°C and 37°C during exponential and stationary phase. As shown in Figure **S8**, expression of a *lon-lacZ* fusion was significantly elevated at 37°C. Moreover, low amounts of the Lon protein were detectable in cell extracts of YPIII at 25°C, whereas significantly higher levels of the protease were found at 37°C. Although analysis of RovA degradation after blockage of protein synthesis clearly demonstrated that thermo- and growth phase-dependent proteolysis of RovA occurs mainly through a superimposed post-translational mechanism (Figures **8** **–** [Fig ppat-1000435-g009] **10**), it is very likely that increased synthesis of the Lon protease at 37°C also contributes to thermo-regulated proteolysis of RovA.

## Discussion

In this study we demonstrate that the global MarR-type virulence regulator RovA of *Yersinia* acts as an intrinsic protein thermometer, that controls its DNA binding activity and regulates its degradation by the ATP-dependent protease Lon, a process which is also subject to growth phase control.

**Figure 11:**
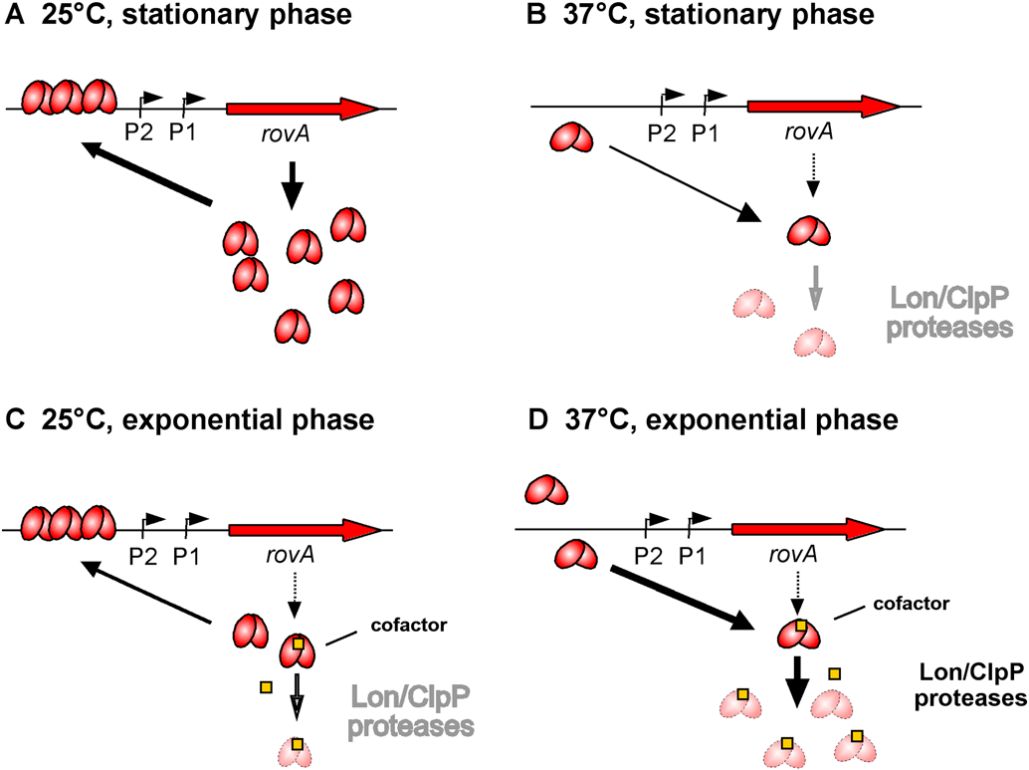
Model of temperature-mediated regulation of *rovA* expression. (A) At moderate temperatures during stationary phase RovA is active and binds cooperatively to its operator sequences upstream of the *rovA* gene. DNA interaction of RovA stimulates transcription by direct activation of the RNA polymerase and antirepression of H-NS mediated silencing. Active conformation and/or DNA-binding block degradation by the Lon and ClpP proteases. (B) Elevated temperature (37°C) induces a conformational change to the inactive, non-DNA-binding form of RovA. This shift in the equilibrium favours the recognition and/or access by the proteases, which leads to an increased degradation of RovA by Lon and to a smaller extent also by ClpP. (C) During exponential phase, a portion of RovA (e.g. non-DNA bound protein) is destablized, e.g. by binding of a cofactor, which increases susceptibility and proteolysis by the proteases at moderate temperatures. (D) At 37°C, partial defolding and inactivation of the RovA protein further improves cofactor binding and/or allow a better access of the proteases, leading to rapid degradation of the regulatory protein.

RovA activity is shown to be strongly dependent on temperature, both *in vivo* and *in vitro*, in such that it binds with higher affinity and enhanced cooperativity to DNA at lower temperatures. According to our model (Figure **11**), the sensor-regulatory activity is based on a conformational adaptation of RovA in response to temperature for regulation of its DNA-binding function. The loss of structured elements upon a temperature shift from 25°C to 37°C strongly supports partial defolding of RovA in this temperature range. This makes RovA less capable of binding DNA in a cooperative manner, and reduces its ability to stimulate the *inv* and *rovA* promoters. Thermo-induced conformational changes are reversible, as α-helicity and DNA-binding capacity of RovA are regained upon cooling to 25°C, and this could be important for the regulatory function of RovA.

To the best of our knowledge only two other bacterial regulators, which belong to a different regulator class are capable of responding directly to temperature: the transcriptional regulator TlpA of *Salmonella enterica* serovar Typhimurium which exhibits homology to KfrA and the SMC proteins involved in plasmid partition or chromosome segregation and the heat shock gene repressor protein RheA of *Streptomyces albus* [34]–[36]. The dimeric TlpA protein forms a long left-handed supercoiled coiled-coil domain in which two subunit α-helices wind around each other and pack their side chains in a “knobs-into-holes” manner [35]. The coiled-coil structure is a versatile and rather flexible motif in mediating protein-protein interactions, and it has been shown that TlpA undergoes a reversible conformational switch in response to temperature changes, leading to alterations between the unfolded monomeric form and the folded DNA-binding coiled-coil oligomeric structure. Also the RheA protein acts as a protein thermometer with shorter coiled-coil domains, and a thermo-induced change in the repressor leads either to an active or inactive form [36]. The structure of the MarR-type regulator RovA is significantly different from TlpA and RheA. Its internal region contains the DNA-binding domain that is predicted to adopt a winged-helix fold. The first N- and the last two C-terminal α-helices appear to form an extensive and well-packed dimer interface, similar to that seen in the structure of other MarR-type regulators. These help to stabilize the formation of dimers with properly positioned DNA-binding segments [23]. This type of dimer formation makes it very unlikely that thermo-sensing of RovA is based on a monomer-to-dimer conversion. In fact, we found no evidence to suggest dissociation of the dimers into monomers at 37°C; in contrast, RovA dimers are still detectable after heating to 95°C in the presence of SDS (H. Tran-Winkler, unpublished results). However, our previous structural-functional analysis showed that even small changes, i.e. single amino acid substitutions in the N-terminal region (L12A, W16A), which have no detectable effect on RovA dimer formation, cause a severe defect in DNA-binding [23]. This suggests that temperature-dependent structural changes within thermo-sensitive elements of the RovA dimer might effect the proper positioning of the DNA-binding segments.

Our studies further demonstrate that the level of RovA in the bacterial cell is not only determined by transcription, but is also subject to growth phase- and temperature-regulated proteolysis. The RovA protein is stable during stationary phase and/or at moderate temperatures (20°C–25°C), but becomes highly unstable during exponential growth at 37°C. Degradation of RovA under these conditions is primarily mediated through the Lon protease, albeit the Clp proteases also appear to participate to a very small extent in the degradation process. Both types of proteases are ATP-dependent and assigned to the AAA^+^ superfamily of ATPases. Lon and ClpP proteases share two common features: (i) access to the proteolytic chamber of the enzyme is usually prohibited to globular proteins, most likely to prevent unrestrained protein degradation, and (ii) they require ATP hydrolysis to unfold and translocate the substrates into the protease chamber. Degradation within the chamber is processive, including sequential rounds of substrate binding, release and rebinding to the proteolytic site, and generates 10–15 amino acid peptides without generation of partially digested protein intermediates [37]–[40]. The ClpP and Lon proteases usually degrade improperly folded or damaged proteins. However, undamaged proteins, in particular short-lived regulatory factors which are implicated in developmental processes, stress resistance and bacterial fitness can also serve as substrates for proteolysis [37].

According to our results, RovA degradation by the Lon protease is clearly temperature-dependent, but alterations of the DNA-binding activity due to thermo-induced conformational changes seems much more critical for thermo-dependent *rovA* expression than the change of RovA stability. For instance, little autoactivation of *rovA* transcription is evident at 37°C, even when RovA is abundant in the absence of the Lon and Clp proteases (Figure **7**). This raises the question of why is RovA also rapidly degraded at 37°C. Thermo-induced structural alterations led to a strong reduction, but did not cause a complete inactivation of the DNA-binding function of RovA (Figures **2**, **3**). Accordingly, degradation of the global regulator might be required to prevent binding to higher affinity sites of RovA within the *Yersinia* genome. Homologous MarR-type regulators were also shown to interact with small metabolites or signalling molecules [41],[42]. In this case, rapid proteolysis of RovA might be essential to prevent sequestration and inactivation of important regulatory components through complex formation with RovA. As RovA proteolysis is also responsible for growth phase regulation of *rovA* and RovA-dependent genes, thermo-dependent alterations of the degradation process might be important to link and coordinate thermoregulation and growth phase control (Figure **11**).

To date very little is known as to what feature identifies transcriptional virulence regulators as substrates of Lon or Clp proteases. Certain peptide motifs of an exposed or unstructured region of a protein, including a few non-polar, aromatic amino acid residues can serve as recognition signal for proteases [31],[43],[44]. Although the major determinants for Lon-mediated proteolysis are frequently found at the N- and C-termini of target proteins [26],[28],[30],[45], it has recently been reported that Lon can obviously also recognize internal tags [44]. In MarR-type regulator proteins, such as RovA, both termini form α-helical structures, which contribute to the formation of the dimer interface [23],[41]. Here we report that transplantation of the N-terminal 96 amino acids of RovA to a normally stable protein confers instability to Lon, but a transfer of the first 42 amino acids does not. Also introduction of a mutation abolishing the DNA-binding function of RovA rendered the protein more susceptible to Lon, whereas presence of a multi-copy plasmid harbouring RovA-binding sites reduced degradation of the protein. This suggests that amino acid residues in the vicinity of the central winged-helix DNA-binding domain comprise the information necessary for Lon binding and/or provides the foundation for degradation by Lon. This finding raises the intriguing possibility that Lon recognition and degradation of RovA is in direct competition with the DNA-binding function of the regulator. In fact, two other substrates, SoxS and the N protein of bacteriophage λ are known to be protected from Lon-mediated degradation when bound to DNA, although their instability is an intrinsic property and does not require an external signal to trigger degradation [46],[47]. From results in this study we know that temperature strongly affects the susceptibility of the RovA protein to Lon-mediated proteolysis. In this regard, it is very likely that the protein degradation signals are normally buried in active RovA and less accessible in RovA-DNA complexes, but become more accessible to the AAA^+^ protease in the non-bound state as a consequence of thermo-induced defolding events (Figure **11**).

Lon-mediated turnover of RovA *in vivo* is likely modulated or regulated by accessory factors that affect either the enzymatic activity of the protease or the conformational state of the target protein. Experiments with an *in vitro* degradation system demonstrated rapid degradation of α-casein by the *Yersinia* Lon protease, but RovA proteolysis was significantly less efficient than proteolysis *in vivo*. In contrast, rapid degradation of RovA could be observed when crude extract of an exponentially grown *lon* mutant strain was added to the *in vitro* system, indicating that an additional component is required for RovA-mediated proteolysis. As RovA degradation is significantly reduced during stationary phase, it seems likely that the postulated accessory component is only present during exponential growth (Figure **11**). Lon-mediated proteolysis might be influenced by interactions with partner proteins, such as adaptors that tether substrates, or chaperones and proteases, which might create or help to expose the degradation signals of RovA. For example, the DnaJ/DnaK/GrpE molecular chaperone system is required to promote formation of certain AAA^+^ protease-substrate complexes [48], and only endoproteolytic cleavage and interaction with the SspB adaptor protein render the transmembrane RseA protein susceptible for ClpXP-mediated proteolysis [49]. Activity of AAA^+^ proteases was further shown to be subject to modulation by cellular compounds, ions and metabolites [50]–[52]. Since MarR-type regulators are frequently modulated by small effector molecules [41],[42], it will also be interesting whether RovA also undergoes effector-induced conformational changes, which could be crucial for growth phase-dependent RovA degradation.

## Materials and Methods

### Bacterial strains and growth conditions

Strains used in this study are listed in Table **S1**. If not indicated otherwise, bacteria were grown at 25°C or 37°C to exponential phase (OD_600_ = 0.4–0.6) or stationary phase in LB broth supplemented with antibiotics as follows: ampicillin 100 µg ml^−1^, chloramphenicol 30 µg ml^−1^, tetracyclin 5 µg ml^−1^, and kanamycin 50 µg ml^−1^.

### Plasmid and strain constructions

Plasmids and primers used in this study are listed in Tables **S1**, **S2** and **S3**. The His_6_-RovA overexpression plasmid pHT95 was cloned by inserting a PCR fragment amplified with primers 1 and 2 into the *Bam*HI/*Pst*I sites of pQE30. pHT105 was constructed by inserting the AatII/AvrII fragment of pZE21, harbouring the P*_tetO-1_* promoter, into pZS*24. Plasmid pHT123 was constructed by inserting a *rovA* ^+^ fragment amplified with primers 3 and 4 into the *Kpn*I and *Cla*I sites of pHT105. Plasmid pHT125 was obtained by inserting a PCR derived fragment amplified with primers 5 and 6 from pTAC3575 into plasmid pGP704 cut with *Sac*I and *Sma*I. For the construction of pKH01 harbouring the *lon-lacZ* fusion, a PCR-derived fragment amplified with primers 7 and 8 from YPIII chromosomal DNA was inserted into pGP20 cut with *Hind*III and *Eco*RI. pKH04 was obtained by inserting the *Kpn*I-*Hind*III *phoA* ^+^ fragment of pHT125 into pHT105. Plasmids pKH08 and pKH26 were obtained by QuikChange mutagenesis (Stratagene) using primer pairs 9/10 and 11/12. The *rovA-lacZ* fusion plasmids pKH15–23 harbouring different portions of the 5′-end of the *rovA* gene were obtained by the insertion of PCR-derived fragments amplified with the upstream primer 13 and the downstream primers 14–21. Plasmid pKH31 was constructed by insertion of a PCR fragment amplified with primers 22 and 23, cloned into the *Kpn*I and *Cla*I sites of pHT105. The *rovA* promoter sequence harbouring the RovA binding sites were amplified by PCR with primers 24 and 25 and cloned into pUC19, generating pKH24. Construction of pKHTS3 was performed by ligation of a PCR fragment amplified with primers 26 and 27 into the *Sac*I and *Pst*I sites of pBAD-HisA. For the construction of pMB113, a *lon* ^+^ PCR fragment, amplified with primers 28 and 29, was inserted into the *Nco*I and *Bgl*II sites of pQE60. For construction of pMB114 the *tet* ^R^ gene was amplified from pACYC184 using primers 30 and 31, and inserted into the *Xho*I and *Sac*I sites of pHT123.

All deletion mutants are derivatives from wild-type strain YPIII and were generated using the RED recombinase system (Derbise *et al.*, 2003) as described in detail in our previous study [24]. For the construction of *E. coli* strain KB2, a kanamycin cassette was integrated into the *hns* locus of *E. coli* and removed as described [53]. The kanamycin or ampicillin resistance gene was amplified with primer pairs 32/33 or 34/35 (Table **S2**). Primers used to amplify 500-bp regions flanking the target genes of *Y. pseudotuberculosis* and primers used for *E. coli* strain KB2 construction are given in Table **S3**.

### Purification of the *Y. pseudotuberculosis* RovA, H-NS, RovM and Lon proteins

All His-tagged proteins were overexpressed in *E. coli* BL21λDE3, native RovA was expressed in *E. coli* strain KB2. Overnight cultures of *E. coli* strains, harbouring the overexpression plasmids pLW1 (*rovA* ^+^), pLW2 (*rovA-his*_6_), pHT95 (*his*_6_-*rovA*), pAKH43 (*rovM* ^+^), pKHTS3 (*his*_6_-*lon*) or pMB113 (*lon-his_6_*) were diluted 1∶100 and grown at 37°C in M9 medium for 2 h. Subsequently, protein synthesis was induced with 100 µM IPTG or 0.02% arabinose (pKHTS3) and grown to an A_600_ of 0.6. Cells overexpressing His-tagged proteins were purified as described [24]. Cells expressing the native RovA protein were resuspended in lysis buffer (10 mM Tris-HCl pH 8.0, 1 mM EDTA, 5 mM DTT, 5% glycerol) and purified on a dsDNA cellulose column (Amersham) using elution buffer (10 mM Tris-HCl pH 8.0, 1 mM EDTA, 5 mM DTT, 5% glycerol, 300 mM NaCl). For the *in vitro* degradation assay, proteins were dialyzed against 50 mM Tris-HCl plus 10 mM MgCl_2_. The purity of the proteins was estimated to be >95%.

### DNA retardation assays

The DNA retardation assays were performed as described [18]. The fragments of the *inv* promoter regions including binding site I, II or both were amplified with the primer pairs 36/37, 38/39 and 40/41. The *rovA* promoter fragments including the RovA or RovM binding sites were amplified with primers 42/43, 42/44 and 45/46, respectively. As a negative control, a 350 bp long fragment from the *E. coli csiD* gene was amplified using primers 47 and 48.

### β-galactosidase and alkaline phosphatase assays

Alkaline phosphatase and β-galactosidase activity were determined as described [24].

### Gel electrophoresis and Western blotting

Preparation and separation of cell extracts as well as Western blotting with polyclonal antibodies directed against RovA, Lon, His-tag or LacZ were performed as described [17],[20].

### CD spectroscopy

0.16 mg/ml purified RovA, 0.4 mg/ml purified RovM and 0.16 mg/ml lysozyme in CD buffer (10 mM NaH_2_PO_4_ pH 8.0, 10 mM NaCl, 5 mM DTT, 1 mM MgCl_2_) were used for CD spectroscopy and CD spectra were recorded on a Jascow J-810 spectrometer using a thermo-stated cell holder. Each spectrum was the result of five successive spectra, each normalized against the CD buffer. Spectra were recorded starting at 25°C. A 10 min equilibrium delay was allowed, after raising the temperature to 37°C. After re-shifting the temperature to 25°C, 2 hrs of equilibration time were allowed. Secondary structure estimation was performed using the analysis software provided by the Jascow J-810 spectrometer, which makes use of the Yang algorithm. Temperature stability was investigated by using a temperature scan from 20°C to 60°C with a temperature slope of 2°C/min at a fixed wavelength of 222 nm. Concentrations of 0.5 mg/ml RovA, 0.2 mg/ml RovM, and 0.5 mg/ml lysozyme were used.

### *In vivo* stability analysis of RovA and RovA-LacZ fusion proteins

Protein biosynthesis of bacterial cultures in exponential or stationary phase was stopped by adding 200 µg/ml chloramphenicol or 50 µg/ml tetracycline. Subsequently, cultures were further incubated at either 25°C or 37°C and samples were taken at indicated time points. Degradation was visualised by Western blotting using a polyclonal anti-RovA or an anti-LacZ antibody as described [20].

### *In vitro* proteolysis of RovA

Purified Lon protease and RovA were dialysed against the reaction buffer (50 mM Tris-HCl pH 8.0; 4 mM DTT, 10 mM MgCl_2_). A concentration of 3 µM Lon protease was mixed with 12.5 µM RovA or α-casein, respectively. ATP was supplied at a concentration of 4 mM and for ATP-regeneration, 80 µg/ml (20 U) creatine kinase and 20 mM creatine phosphate were added. Reaction mixtures were separated and incubated at either 25°C or 37°C. After specified time points the reaction was stopped by adding SDS-sample buffer and heat denaturation at 95°C for 5 min. Degradation of RovA and α-casein was visualised by SDS-PAGE and coomassie staining. To test whether an additional component is required for *in vitro* proteolysis, BL21λDE3 (*lon* ^−^) pLW1 was grown to A_600_ of 0.6 at 25°C and 37°C, resuspended in reaction buffer and lysed using a French Press (120 000 psi). A concentration of 3 µM purified Lon protease was given to the extracts, with or without addition of ATP and the ATP-regeneration system. Degradation of the RovA protein in the sample was analyzed as described above.

## Supporting Information

Figure S1 Predicted structural model of RovA. (A) Predicted secondary structure of RovA matched with the secondary structure of homologous proteins MarR, MexR and SlyAEF based on X-ray crystallographic data. (B) The RovA dimer structure is illustrated as proposed in our previous study, addressing the functional organization of RovA [23]. The α-helices, the β-sheets of the winged-helix DNA-binding region and the termini of one monomer (orange) are indicated.
(3.91 MB PDF)

Figure S2 Conformational analysis of lysozyme using CD spectroscopy. (A) CS spectra, Δε (M^−1^ cm^−1^) versus wavelength of lysozyme (0.16 mg/ml) as function of temperature (25°C, light grey; 37°C dark grey; 37°C and cooling to 25°C medium grey). (B) Thermal stability of lysozyme. The temperature of the lysozyme solution was increased from 20°C to 60°C with a temperature slope of 2°C/min. The denaturation curve was recorded at a fixed wavelength of μ = 222 nm. The melting point (T_m_) was calculated using the Jascow spectra analysis software.
(0.73 MB PDF)

Figure S3 Stability of RovM during exponential and stationary phase at 25°C and 37°C. Cultures of *Y. pseudotuberculosis* YPIII (A–B) and *E. coli* DH5aZ1 pKH31 (C–F) were grown overnight or to exponential phase (OD_600_ = 0.3–0.4) at 25°C before chloramphenicol (200 µg ml^−1^) was added. The cultures were divided and incubated at 25°C or 37°C for additional 90 min. Aliquots of the cultures were removed at the indicated times thereafter, whole cell extracts from identical numbers of bacteria were prepared and analyzed by Western blotting with a polyclonal antibody directed against RovM. Whole cell extracts from the *rovM* mutant strain YP72 grown overnight at 25°C were used as control. Protein bands were quantified using the BioRad analysis software ‘Quantity One’ 4.6.2 and set into relation to sampling at time point zero (diamond: 25°C, stationary phase; triangle: 37°C, stationary phase; square: 25°C, exponential phase; circle: 37°C, exponential phase).
(1.11 MB PDF)

Figure S4 Quantification of RovA degradation. (A) *In vivo* degradation of RovA in YPIII (wt), YP63 (*clpP*−), YP67 (*lon*−) and YP68 (*clpP/lon*−) at 25°C and 37°C (see also Fig. 8). (B) *In vivo* degradation of RovA-LacZ fusion proteins in YPIII (wt) or YP67 (*lon*−) at 37°C (see also Fig. 9). (C) *In vitro* degradation at 25°C and 37°C of casein (left panel) and RovA (middle panel) with or without Lon protease. *In vitro* degradation of RovA in the presence of whole cell extract with or without Lon protease and ATP (right panel) (see also Fig. 10).
(1.10 MB PDF)

Figure S5 Stability of RovA during stationary phase at 25°C and 37°C in *Y. pseudotuberculosis* YPIII and the *clpP, lon*, and *clp/lon* deletion mutants. Cultures of *Y. pseudotuberculosis* strain YPIII (wt) and the *clpP, lon*, and *clp/lon* deletion mutants were grown overnight at 25°C before chloramphenicol (200 µg ml^−1^) was added to stop protein synthesis. The cultures were divided and incubated at 25°C or 37°C for additional 10 h. Aliquots of the cultures were removed at the indicated times thereafter, whole cell extracts from identical number of bacteria were prepared and analyzed by Western blotting with a polyclonal antibody directed against RovA. Whole cell extracts from the *rovA* mutant strain YP3 grown overnight at 25°C were used as control. A prestained molecular weight marker is loaded on the left.
(1.17 MB PDF)

Figure S6 DNA-binding protects RovA proteolysis. (A) Stability of RovA during exponential phase at 37°C in *Y. pseudotuberculosis* YPIII pUC19 and YPIII pKH24, harbouring the RovA binding sites of the *rovA* promoter. (B) Stability of the RovA mutant protein RovAE71K and the RovA wildtype protein expressed by YPIII pKH26 and YPIII pHT123, respectively during the exponential phase at 37°C. Cultures of the *Y. pseudotuberculosis* strains were grown to exponential phase (OD600 = 0.3−0.4) at 25°C before chloramphenicol (200 µg ml^−1^) was added to stop protein synthesis. The cultures were shifted to 37°C for additional 90 min. Aliquots of the cultures were removed at the indicated times, whole cell extracts from identical numbers of bacteria were prepared and analyzed by Western blotting with a polyclonal antibody directed against RovA. The *rovA* knock-out strain YP3 was used as a control. A prestained molecular weight marker is loaded on the left.
(1.24 MB PDF)

Figure S7 *In vitro* degradation of RovA. Whole cell extracts of BL21 (*lon*-) were prepared and added to purified RovA, and incubated at 37°C in the presence of ATP without Lon (A) or incubated at 25°C in the presence of purified Lon and ATP (B). The degradation reaction was performed as described in Materials and Methods. An aliquot was removed at indicated times, separated on 15% SDS-gels, and visualised by Western blotting with an anti-RovA antibody. * indicates protein bands that interact non-specifically with the RovA antibody used for loading control.
(1.28 MB PDF)

Figure S8 Temperature- and stationary phase-dependent regulation of *lon* expression. Expression of *lon* was analyzed in *Y. pseudotuberculosis* YPIII (wt) harbouring a *lon-lacZ* fusion in exponential and stationary phase cells grown at 25°C or 37°C. β-galactosidase activity was determined and is given in µmol min^−1^ mg^−1^ for comparison. The data represent the average SD from at least three different experiments each done in duplicate (upper panel). Furthermore, whole cell extracts of equal amounts of the bacteria were prepared, separated by SDS-PAGE, and visualized by immunoblotting using a polyclonal antibody directed against Lon of *E. coli*. Whole cell extracts of a *Y. pseudotuberculosis lon* mutant were used as controls. A prestained molecular weight marker is loaded on the left (lower panel).
(1.40 MB PDF)

Table S1 Bacterial strains and plasmids.
(0.08 MB PDF)

Table S2 Oligonucleotides used in this study.
(0.06 MB PDF)

Table S3 Primers used for the generation of deletion mutants.
(0.05 MB PDF)
