# Supplementary material for: Intrinsic Thermal Sensing Controls Proteolysis of Yersinia Virulence Regulator RovA
Source: PLoS Pathog. 2009 May 15;5(5):e1000435. doi: 10.1371/journal.ppat.1000435 (PMC2676509; doi:10.1371/journal.ppat.1000435)
Supplement: Figure S1 — Predicted structural model of RovA. (A) Predicted secondary structure of RovA matched with the secondary structure of homologous proteins MarR, MexR and SlyAEF based on X-ray crystallographic data. (B) The RovA dimer structure is illustrated as proposed in our previous study, addressing the functional organization of RovA [23]. The α-helices, the β-sheets of the winged-helix DNA-binding region and the termini of one monomer (orange) are indicated. (3.91 MB PDF) [file ppat.1000435.s001.pdf]

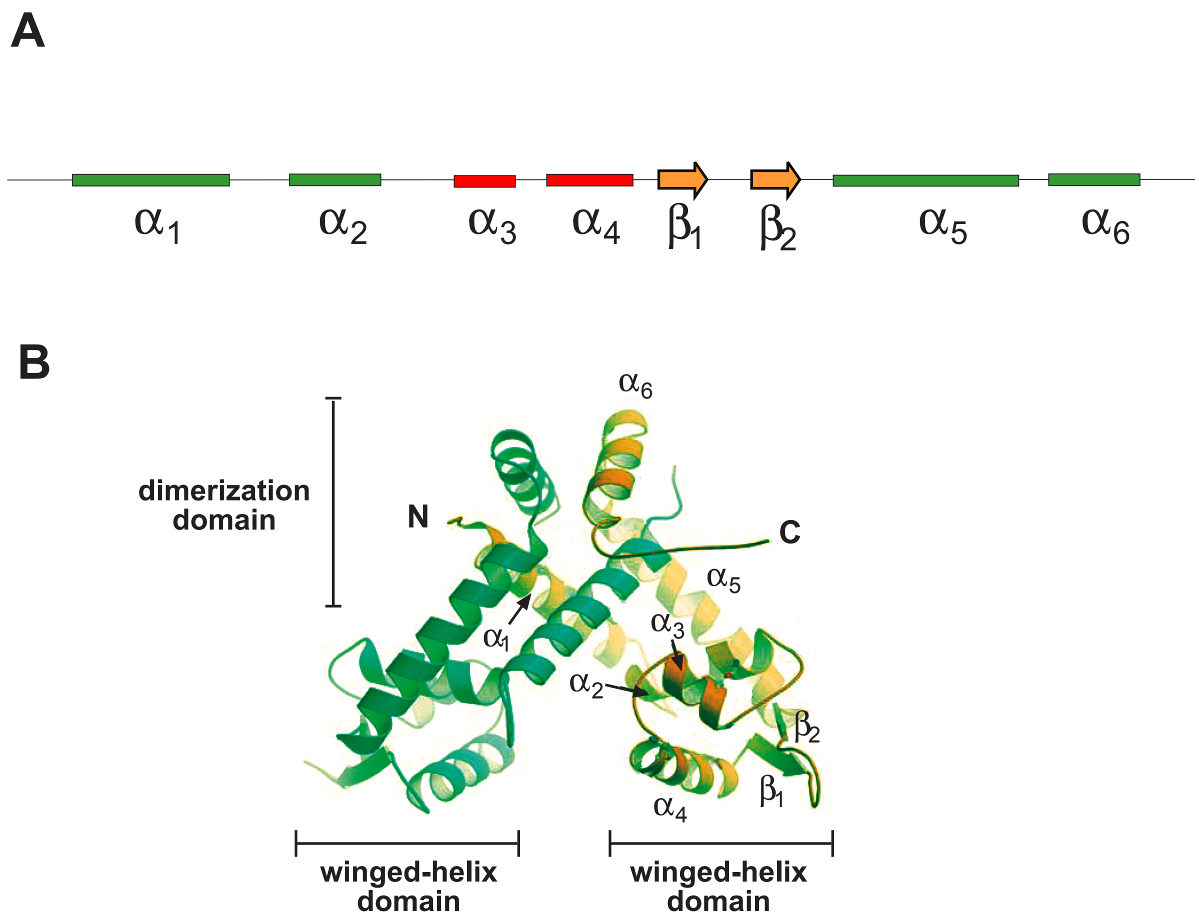

Supplementary Figure S1

Predicted structural model of RovA. (A) Predicted secondary structure of RovA matched with the secondary structure of homologous proteins MarR, MexR and SlyA*EF*based on X-ray crystallographic data. (B) The RovA dimer structure is illustrated as proposed in our previous study, addressing the functional organization of RovA (Tran *et al.* 2005, J. Biol. Chem. 280: 42423-42432). The α-helices, the β-sheets of the winged-helix DNA-binding region and the termini of one monomer (orange) are indicated.

Herbst *et al.* 2009
