# Supplementary material for: Intrinsic Thermal Sensing Controls Proteolysis of Yersinia Virulence Regulator RovA
Source: PLoS Pathog. 2009 May 15;5(5):e1000435. doi: 10.1371/journal.ppat.1000435 (PMC2676509; doi:10.1371/journal.ppat.1000435)
Supplement: Figure S2 — Conformational analysis of lysozyme using CD spectroscopy. (A) CS spectra, Δε (M−1 cm−1) versus wavelength of lysozyme (0.16 mg/ml) as function of temperature (25°C, light grey; 37°C dark grey; 37°C and cooling to 25°C medium grey). (B) Thermal stability of lysozyme. The temperature of the lysozyme solution was increased from 20°C to 60°C with a temperature slope of 2°C/min. The denaturation curve was recorded at a fixed wavelength of μ = 222 nm. The melting point (Tm) was calculated using the Jascow spectra analysis software. (0.73 MB PDF) [file ppat.1000435.s002.pdf]

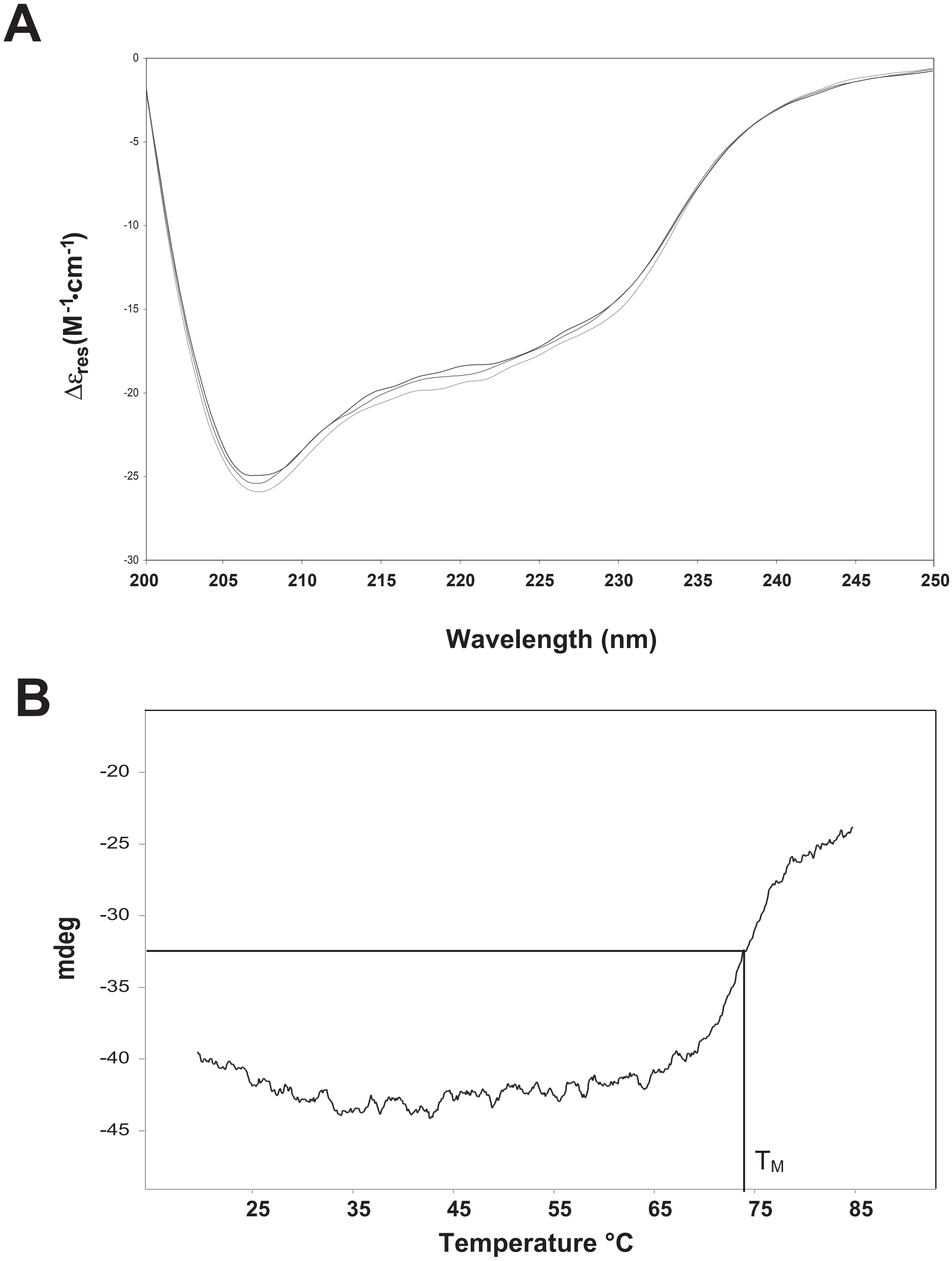

Supplementary Figure S2

Conformational analysis of lysozyme using CD spectroscopy. (A) CS spectra, $\Delta\varepsilon$ ($M^{-1}cm^{-1}$) versus wavelength of lysozyme (0.16 mg/ml) as function of temperature (25°C, light grey; 37°C dark grey; 37°C and cooling to 25°C medium grey). (B) Thermal stability of lysozyme. The temperature of the lysozyme solution was increased from 20°C to 60°C with a temperature slope of 2°C/min. The denaturation curve was recorded at a fixed wavelength of µ=222 nm. The melting point (T_M) was calculated using the Jascow spectra analysis software.
