# Supplementary material for: Intrinsic Thermal Sensing Controls Proteolysis of Yersinia Virulence Regulator RovA
Source: PLoS Pathog. 2009 May 15;5(5):e1000435. doi: 10.1371/journal.ppat.1000435 (PMC2676509; doi:10.1371/journal.ppat.1000435)
Supplement: Figure S3 — Stability of RovM during exponential and stationary phase at 25°C and 37°C. Cultures of Y. pseudotuberculosis YPIII (A–B) and E. coli DH5aZ1 pKH31 (C–F) were grown overnight or to exponential phase (OD600 = 0.3–0.4) at 25°C before chloramphenicol (200 µg ml−1) was added. The cultures were divided and incubated at 25°C or 37°C for additional 90 min. Aliquots of the cultures were removed at the indicated times thereafter, whole cell extracts from identical numbers of bacteria were prepared and analyzed by Western blotting with a polyclonal antibody directed against RovM. Whole cell extracts from the rovM mutant strain YP72 grown overnight at 25°C were used as control. Protein bands were quantified using the BioRad analysis software ‘Quantity One’ 4.6.2 and set into relation to sampling at time point zero (diamond: 25°C, stationary phase; triangle: 37°C, stationary phase; square: 25°C, exponential phase; circle: 37°C, exponential phase). (1.11 MB PDF) [file ppat.1000435.s003.pdf]

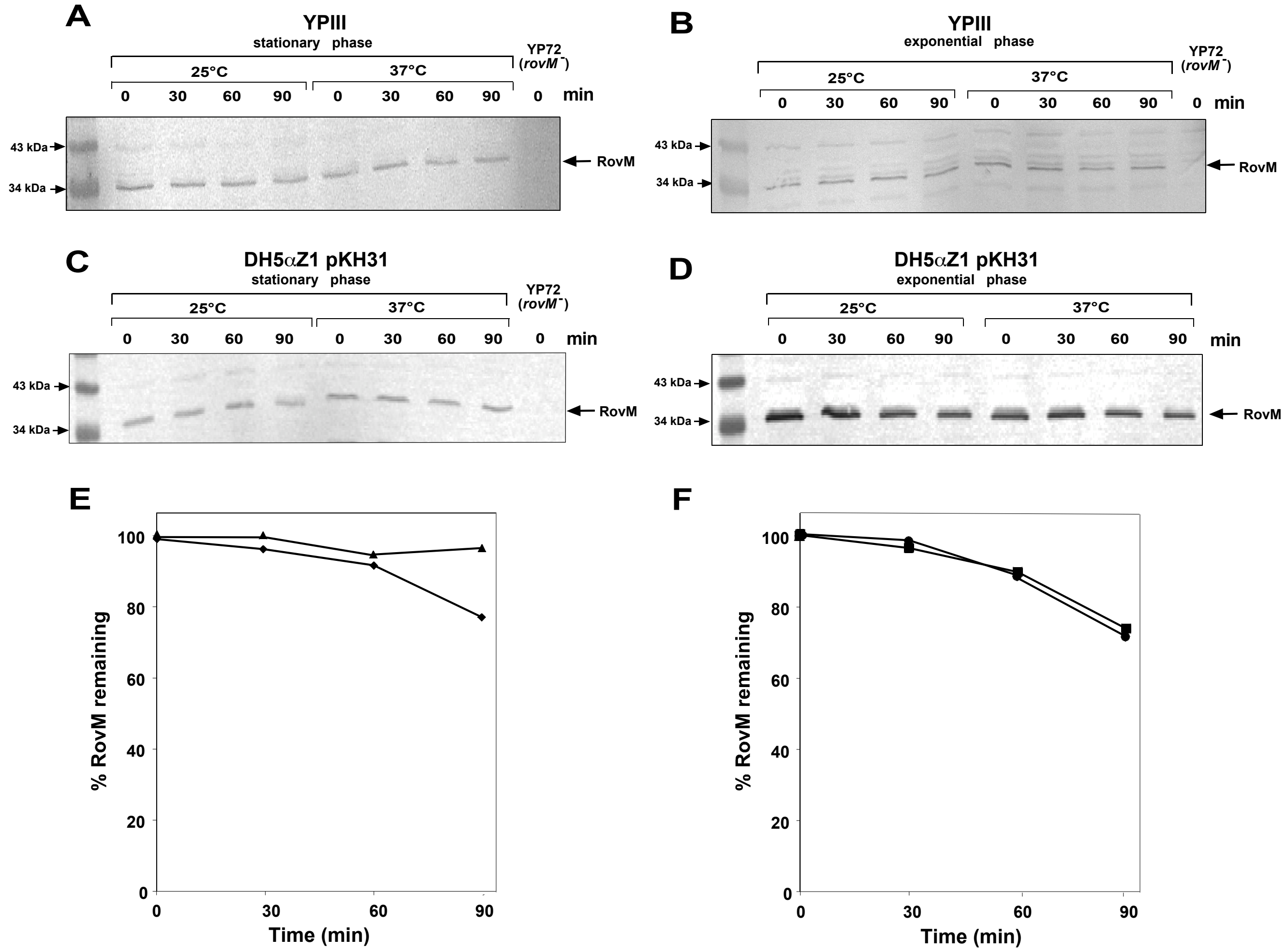

Supplementary Fig. S3

Stability of RovM during exponential and stationary phase at 25°C and 37°C. Cultures of *Y. pseudotuberculosis* YPIII (A-B) and *E. coli* DH5αZ1 pKH31 (C-F) were grown overnight or to exponential phase (OD_{600}= 0.3-0.4) at 25°C before chloramphenicol (200 µg ml^{-1}) was added. The cultures were divided and incubated at 25°C or 37°C for additional 90 min. Aliquots of the cultures were removed at the indicated times thereafter, whole cell extracts from identical numbers of bacteria were prepared and analyzed by Western blotting with a polyclonal antibody directed against RovM. Whole cell extracts from the *rovM* mutant strain YP72 grown overnight at 25°C were used as control. Protein bands were quantified using the BioRad analysis software 'Quantity One' 4.6.2 and set into relation to sampling at time point zero (◆ 25°C, stationary phase; ▲ 37°C, stationary phase; ■ 25°C, exponential phase; ● 37°C, exponential phase).

Herbst *et al.* 2009
