# Supplementary material for: Intrinsic Thermal Sensing Controls Proteolysis of Yersinia Virulence Regulator RovA
Source: PLoS Pathog. 2009 May 15;5(5):e1000435. doi: 10.1371/journal.ppat.1000435 (PMC2676509; doi:10.1371/journal.ppat.1000435)
Supplement: Figure S4 — Quantification of RovA degradation. (A) In vivo degradation of RovA in YPIII (wt), YP63 (clpP−), YP67 (lon−) and YP68 (clpP/lon−) at 25°C and 37°C (see also Fig. 8). (B) In vivo degradation of RovA-LacZ fusion proteins in YPIII (wt) or YP67 (lon−) at 37°C (see also Fig. 9). (C) In vitro degradation at 25°C and 37°C of casein (left panel) and RovA (middle panel) with or without Lon protease. In vitro degradation of RovA in the presence of whole cell extract with or without Lon protease and ATP (right panel) (see also Fig. 10). (1.10 MB PDF) [file ppat.1000435.s004.pdf]

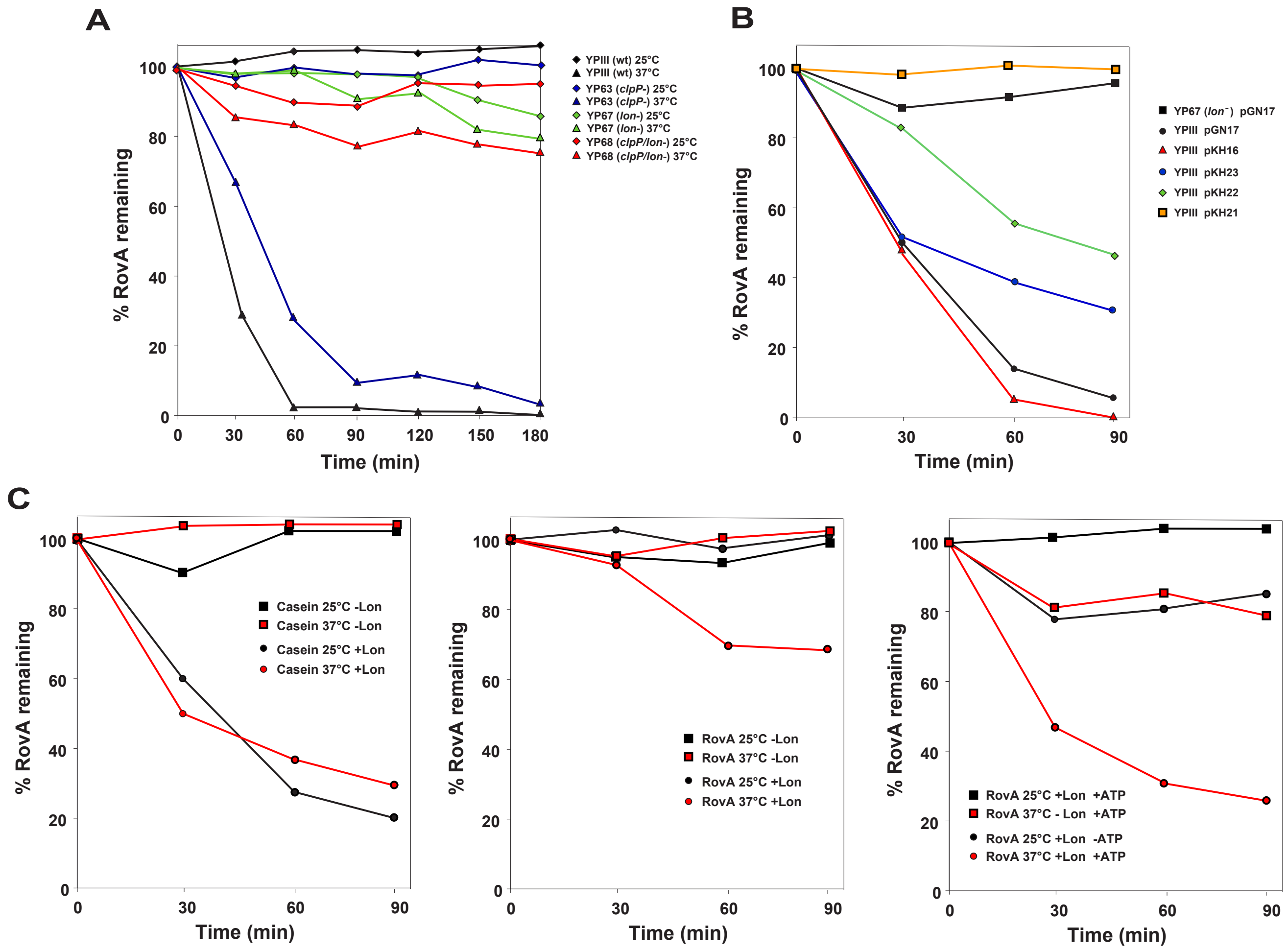

Supplementary Fig. S4

Quantification of RovA degradation. (**A**) *In vivo* degradation of RovA in YPIII (wt), YP63 (*clpP*-), YP67 (*lon*-) and YP68 (*clpP/lon*-)at 25°C and 37°C (see also Fig. 8). (**B**) *In vivo* degradation of RovA-LacZ fusion proteins in YPIII (wt) or YP67 (*lon*-) at 37°C (see also Fig. 9). (**C**) *In vitro* degradation at 25°C and 37°C of casein (left panel) and RovA (middle panel) with or without Lon protease. *In vitro* degradation of RovA in the presence of whole cell extract with or without Lon protease and ATP (right panel) (see also Fig. 10).

Herbst *et al.* 2009
