# Supplementary material for: Intrinsic Thermal Sensing Controls Proteolysis of Yersinia Virulence Regulator RovA
Source: PLoS Pathog. 2009 May 15;5(5):e1000435. doi: 10.1371/journal.ppat.1000435 (PMC2676509; doi:10.1371/journal.ppat.1000435)
Supplement: Figure S5 — Stability of RovA during stationary phase at 25°C and 37°C in Y. pseudotuberculosis YPIII and the clpP, lon, and clp/lon deletion mutants. Cultures of Y. pseudotuberculosis strain YPIII (wt) and the clpP, lon, and clp/lon deletion mutants were grown overnight at 25°C before chloramphenicol (200 µg ml−1) was added to stop protein synthesis. The cultures were divided and incubated at 25°C or 37°C for additional 10 h. Aliquots of the cultures were removed at the indicated times thereafter, whole cell extracts from identical number of bacteria were prepared and analyzed by Western blotting with a polyclonal antibody directed against RovA. Whole cell extracts from the rovA mutant strain YP3 grown overnight at 25°C were used as control. A prestained molecular weight marker is loaded on the left. (1.17 MB PDF) [file ppat.1000435.s005.pdf]

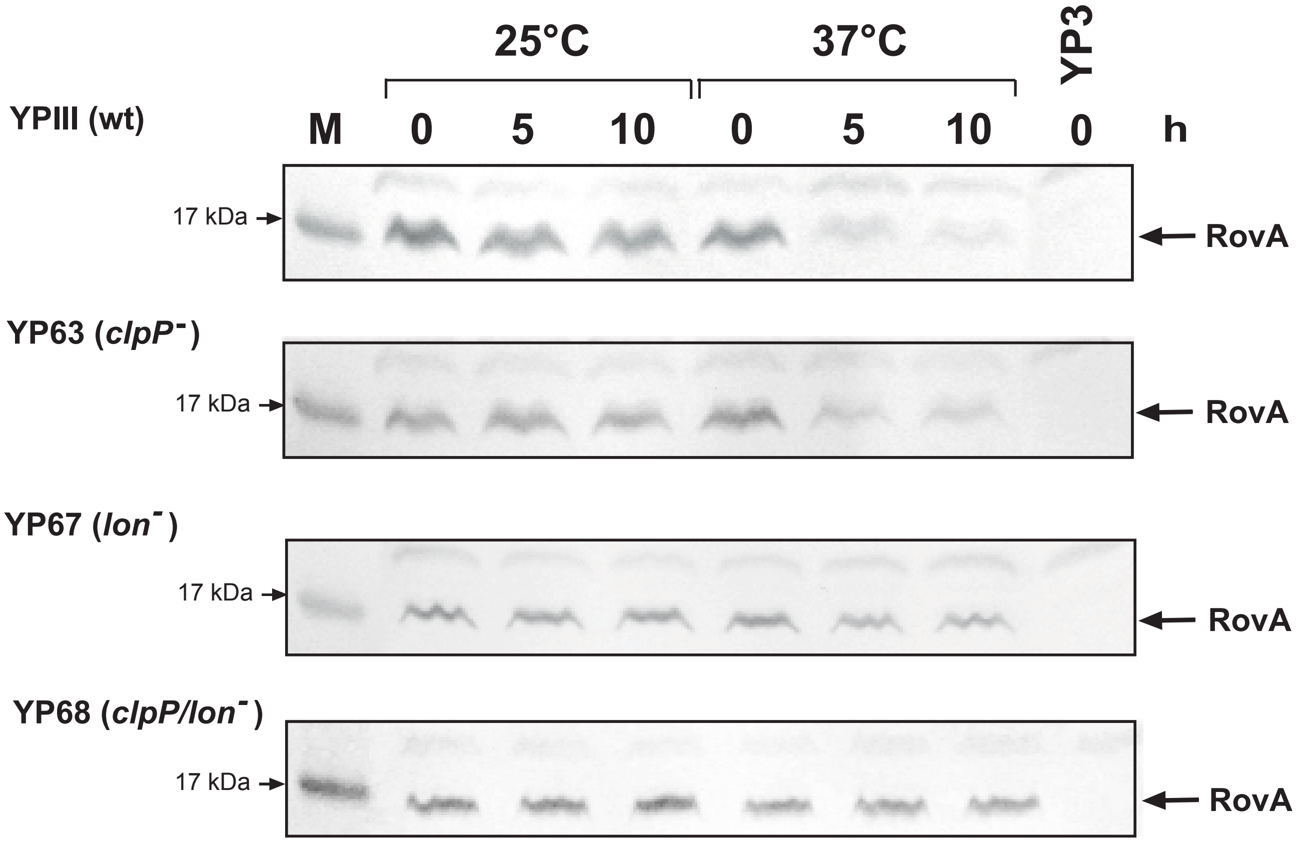

Supplementary Fig. S5

Stability of RovA during stationary phase at 25°C and 37°C in *Y. pseudotuberculosis* YPIII and the *clpP, lon*, and *clp/lon* deletion mutants. Cultures of *Y. pseudotuberculosis* strain YPIII (wt) and the *clpP, lon*, and *clp/lon* deletion mutants were grown overnight at 25°C before chloramphenicol (200 µg ml^{-1}) was added to stop protein synthesis. The cultures were divided and incubated at 25°C or 37°C for additional 10 h. Aliquots of the cultures were removed at the indicated times thereafter, whole cell extracts from identical number of bacteria were prepared and analyzed by Western blotting with a polyclonal antibody directed against RovA. Whole cell extracts from the *rovA* mutant strain YP3 grown overnight at 25°C were used as control. A prestained molecular weight marker is loaded on the left.

Herbst *et al.* 2009
