# Supplementary material for: Intrinsic Thermal Sensing Controls Proteolysis of Yersinia Virulence Regulator RovA
Source: PLoS Pathog. 2009 May 15;5(5):e1000435. doi: 10.1371/journal.ppat.1000435 (PMC2676509; doi:10.1371/journal.ppat.1000435)
Supplement: Figure S6 — DNA-binding protects RovA proteolysis. (A) Stability of RovA during exponential phase at 37°C in Y. pseudotuberculosis YPIII pUC19 and YPIII pKH24, harbouring the RovA binding sites of the rovA promoter. (B) Stability of the RovA mutant protein RovAE71K and the RovA wildtype protein expressed by YPIII pKH26 and YPIII pHT123, respectively during the exponential phase at 37°C. Cultures of the Y. pseudotuberculosis strains were grown to exponential phase (OD600 = 0.3−0.4) at 25°C before chloramphenicol (200 µg ml−1) was added to stop protein synthesis. The cultures were shifted to 37°C for additional 90 min. Aliquots of the cultures were removed at the indicated times, whole cell extracts from identical numbers of bacteria were prepared and analyzed by Western blotting with a polyclonal antibody directed against RovA. The rovA knock-out strain YP3 was used as a control. A prestained molecular weight marker is loaded on the left. (1.24 MB PDF) [file ppat.1000435.s006.pdf]

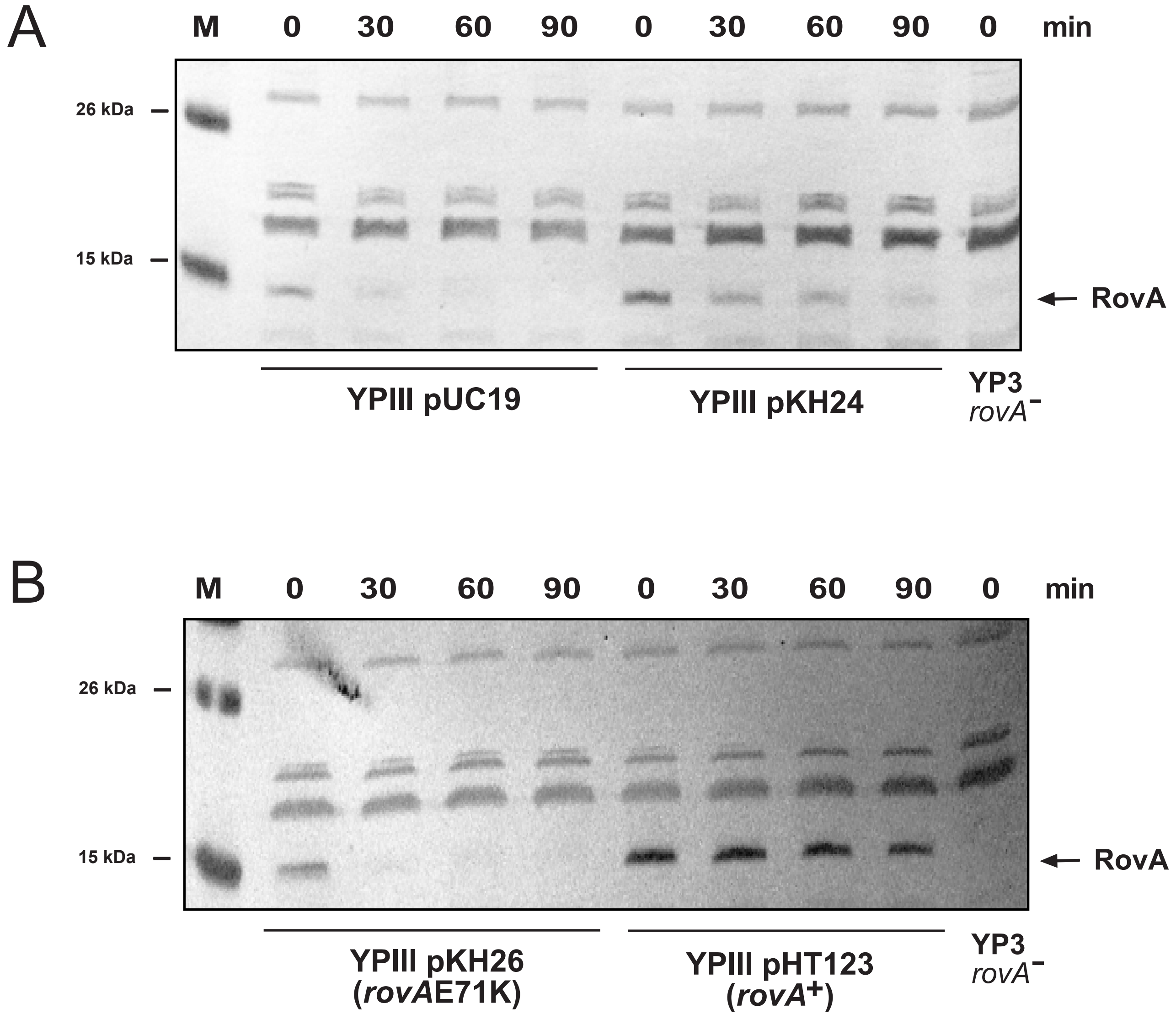

Supplementary Fig. S6

DNA-binding protects RovA proteolysis. (**A**) Stability of RovA during exponential phase at 37°C in *Y. pseudotuberculosis* YPIII pUC19 and YPIII pKH24, harbouring the RovA binding sites of the *rovA* promoter. (**B**) Stability of the RovA mutant protein RovAE71K and the RovA wildtype protein expressed by YPIII pKH26 and YPIII pHT123, respectively during the exponential phase at 37°C. Cultures of the *Y. pseudotuberculosis* strains were grown to exponential phase (OD_{600}= 0.3-0.4) at 25°C before chloramphenicol (200 µg ml^{-1}) was added to stop protein synthesis. The cultures were shifted to 37°C for additional 90 min. Aliquots of the cultures were removed at the indicated times, whole cell extracts from identical numbers of bacteria were prepared and analyzed by Western blotting with a polyclonal antibody directed against RovA. The *rovA* knock-out strain YP3 was used as a control. A prestained molecular weight marker is loaded on the left.

Herbst *et al.* 2009
