# Supplementary material for: Intrinsic Thermal Sensing Controls Proteolysis of Yersinia Virulence Regulator RovA
Source: PLoS Pathog. 2009 May 15;5(5):e1000435. doi: 10.1371/journal.ppat.1000435 (PMC2676509; doi:10.1371/journal.ppat.1000435)
Supplement: Figure S7 — In vitro degradation of RovA. Whole cell extracts of BL21 (lon-) were prepared and added to purified RovA, and incubated at 37°C in the presence of ATP without Lon (A) or incubated at 25°C in the presence of purified Lon and ATP (B). The degradation reaction was performed as described in Materials and Methods. An aliquot was removed at indicated times, separated on 15% SDS-gels, and visualised by Western blotting with an anti-RovA antibody. * indicates protein bands that interact non-specifically with the RovA antibody used for loading control. (1.28 MB PDF) [file ppat.1000435.s007.pdf]

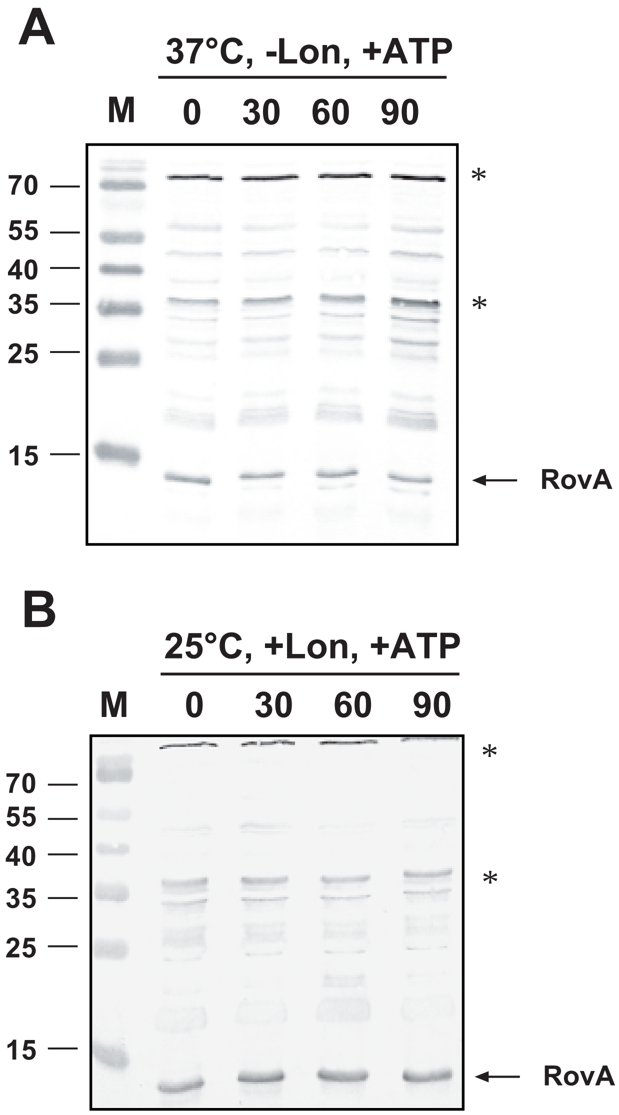

Supplementary Fig. S7

In vitro degradation of RovA. Whole cell extracts of BL21 (*lon*-) were prepared and added to purified RovA, and incubated at 37°C in the presence of ATP without Lon (**A**) or incubated at 25°C in the presence of purified Lon and ATP (**B**). The degradation reaction was performed as described in Material and Methods. An aliquot was removed at indicated times, separated on 15% SDS-gels, and visualised by Western blotting with an anti-RovA antibody. * indicates protein bands that interact non-specifically with the RovA antibody used for loading control.

Herbst *et al.* 2009
