# Supplementary material for: Intrinsic Thermal Sensing Controls Proteolysis of Yersinia Virulence Regulator RovA
Source: PLoS Pathog. 2009 May 15;5(5):e1000435. doi: 10.1371/journal.ppat.1000435 (PMC2676509; doi:10.1371/journal.ppat.1000435)
Supplement: Figure S8 — Temperature- and stationary phase-dependent regulation of lon expression. Expression of lon was analyzed in Y. pseudotuberculosis YPIII (wt) harbouring a lon-lacZ fusion in exponential and stationary phase cells grown at 25°C or 37°C. β-galactosidase activity was determined and is given in µmol min−1 mg−1 for comparison. The data represent the average SD from at least three different experiments each done in duplicate (upper panel). Furthermore, whole cell extracts of equal amounts of the bacteria were prepared, separated by SDS-PAGE, and visualized by immunoblotting using a polyclonal antibody directed against Lon of E. coli. Whole cell extracts of a Y. pseudotuberculosis lon mutant were used as controls. A prestained molecular weight marker is loaded on the left (lower panel). (1.40 MB PDF) [file ppat.1000435.s008.pdf]

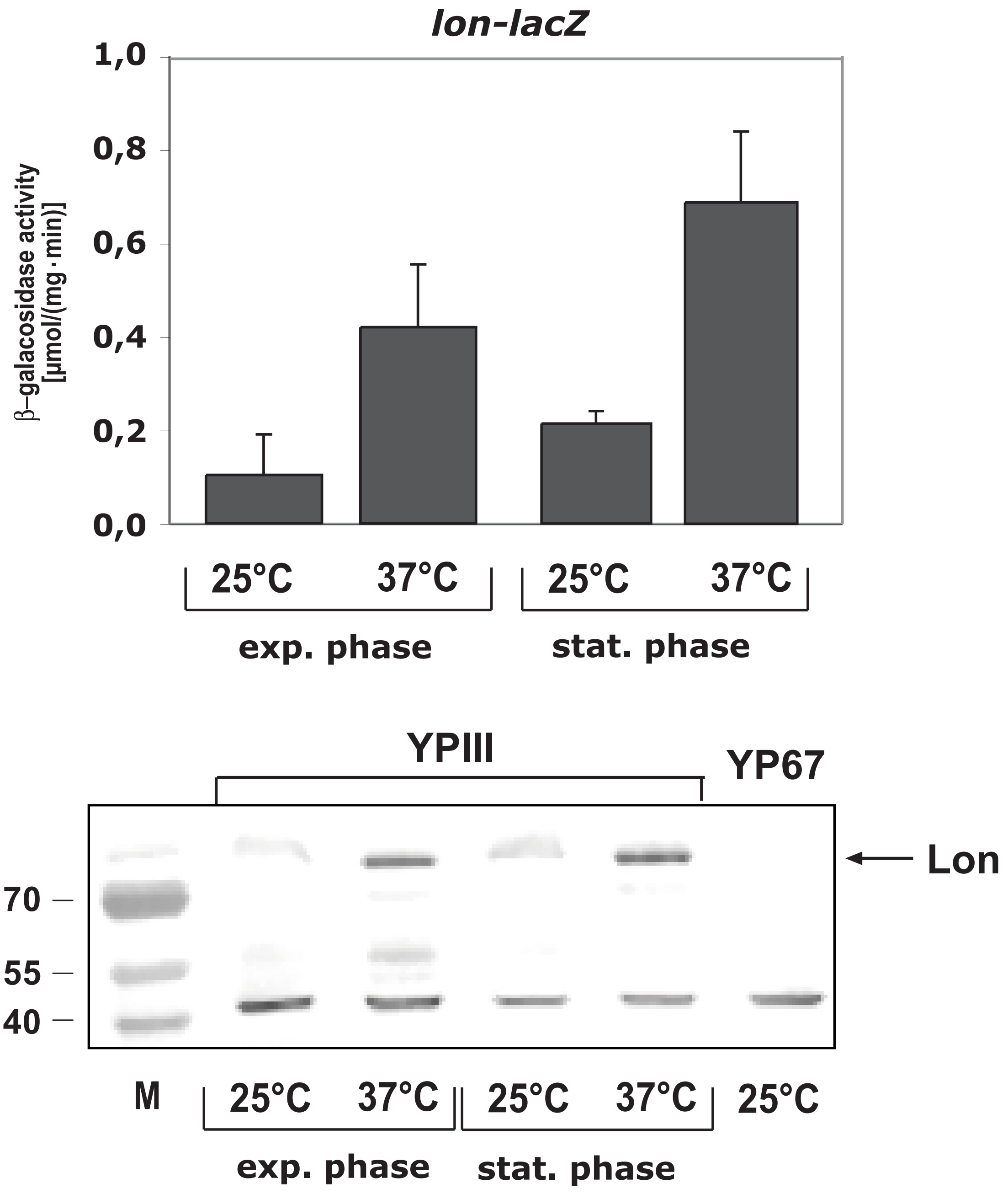

Supplementary Fig. S8

Temperature- and stationary phase-dependent regulation of *lon* expression. Expression of *lon* was analyzed in *Y. pseudotuberculosis* YPIII (wt) harbouring a *lon-lacZ* fusion in exponential and stationary phase cells grown at 25°C or 37°C. β-galactosidase activity was determined and is given in µmol min^{-1} mg^{-1} for comparison. The data represent the average SD from at least three different experiments each done in duplicate (upper panel). Furthermore, whole cell extracts of equal amounts of the bacteria were prepared, separated by SDS-PAGE, and visualized by immunoblotting using a polyclonal antibody directed against Lon of *E. coli*. Whole cell extracts of a *Y. pseudotuberculosis lon* mutant were used as controls. A prestained molecular weight marker is loaded on the left (lower panel).

Herbst *et al.* 2009
