# Supplementary material for: Intrinsic Thermal Sensing Controls Proteolysis of Yersinia Virulence Regulator RovA
Source: PLoS Pathog. 2009 May 15;5(5):e1000435. doi: 10.1371/journal.ppat.1000435 (PMC2676509; doi:10.1371/journal.ppat.1000435)
Supplement: Table S1 — Bacterial strains and plasmids. (0.08 MB PDF) [file ppat.1000435.s009.pdf]

Table S1. Bacterial strains and plasmids.

Strains, Plasmids	*Description*	*Source and reference*
**Bacterial strains**		
E. coli K-12		
CC118λpir	F^{-} Δ(*ara-leu*)7697 Δ(*lacZ*)74 Δ(*phoA*)20 *araD139 galE galK thi rpsE rpoB arfE*^{am} *recA1*, λ*pir*	(Manoil and Beckwith, 1986)
BL21λDE3	F^{-} *gal met r*$^{-}$*m*$^{-}$ *lon hsdS* λ_{Lys}p*lac*UV5-T7 gene1 p*lacI*^{q} *lacI*	(Studier and Moffatt, 1986)
DH5αZ1	(ϕ80d *lacZ*ΔM15) *endA1 recA1 hsdR17 supE44 thi-1, gyrA*	(Lutz and Bujard, 1997)
KB2	BL21λDE3, *hns*$^{-}$	this study
Y. pseudotuberculosis		
YPIII	pIB1, wild type	(Bolin *et al.*, 1982)
YP3	pIB1, *rovA*::Tn*10*(60)[a]; Cm^{R}	(Nagel *et al.*, 2001)
YP63	pIB1, Δ*clpP*; Kn^{R}	this study
YP67	pIB1, Δ*lon*; Kn^{R}	this study
YP68	pIB1, Δ*clpP*, Δ*lon*; Ap^{R}, Kn^{R}	this study
**Plasmids**		
pACYC184	cloning vector, p15A, Cm^{R}, Tet^{R}	(Chang and Cohen, 1978)
pAKH43	pET28a, *rovM*$^{+}$, Kn^{R}	(Heroven and Dersch, 2006)
pAKH47	pGP20, *rovA-lacZ*(17)[b], Tet^{R}	(Heroven and Dersch, 2006)
pBAD-HisA	overexpression vector, Ap^{R}	Invitrogen
pCP20	pSC101 derivative on TS, *bla cat* cI857 IPR *flp*	(Datsenko and Wanner, 2000)
pET28a	overexpression vector, Kn^{R}	Novagen
pGP704	R6K cloning plasmid, Ap^{R}	(Miller and Falkow, 1988)
pGN17	pACYC184, *rovA-lacZ*(127)[b], Cm^{R}	(Heroven *et al.*, 2004)
pGN25	pACYC184, -547 bp *rovA* upstream region *rovA-lacZ*(17)[b], Cm^{R}	(Heroven *et al.*, 2004)
pHT95	pQE30, *his*$_{6}$-*rovA*$^{+}$,	this study
pHT105	pZS*24, $P_{LtetO-1}$, Kn^{R}	this study
pHT123	pHT105, $P_{LtetO-1}$::*rovA*$^{+}$, Kn^{R}	this study
pHT125	pGP704, *phoA*$^{+}$, Ap^{R}	this study
pJG23	ColEI cloning vector, *csiD*$^{+}$, Ap^{R}	J. Germer
pKD4	R6K derivative *bla* FRT Kan^{R} FRT PS1 PS2	(Datsenko and Wanner, 2000)

pKD46	pSC101 derivative on TS, P_{BAD} *gam bet exo*	(Datsenko and Wanner, 2000)
pKH01	pGP20, *lon-lacZ*(6)[b], Tet^R	this study
pKH04	pZS-21, $P_{LtetO-1}$::*phoA*$^+$, Kn^R	this study
pKH08	pHT123, $P_{LtetO-1}$::*his*$_6$-*rovA*$^+$, Kn^R	this study
pKH15	pACYC184, *rovA-lacZ*(23)[b], Cm^R	this study
pKH16	pACYC184, *rovA-lacZ*(106)[b], Cm^R	this study
pKH17	pACYC184, *rovA-lacZ*(101)[b], Cm^R	this study
pKH18	pACYC184, *rovA-lacZ*(117)[b], Cm^R	this study
pKH20	pACYC184, *rovA-lacZ*(26)[b], Cm^R	this study
pKH21	pACYC184, *rovA-lacZ*(42)[b], Cm^R	this study
pKH22	pACYC184, *rovA-lacZ*(74)[b], Cm^R	this study
pKH23	pACYC184, *rovA-lacZ*(96)[b], Cm^R	this study
pKH24	pUC19, P*rovA*, Ap^R	this study
pKH26	pHT123, $P_{LtetO-1}$::*rovA*(E71K), Kn^R	this study
pKH31	pHT105, $P_{LtetO-1}$::*rovM*$^+$, Kn^R	this study
pKHTS3	pBAD-HisA, *lon*$^+$, Ap^R,	this study
pKOBEG-*sacB*	recombination vector, *sacB*$^+$, Cm^R	(Derbise *et al.*, 2003)
pLW1	pET28a, *rovA*$^+$, Kn^R	L. Winkler
pLW2	pET28a, *rovA-his*$_6^+$, Kn^R	(Tran *et al.*, 2005)
pMB113	pQE60, *lon-his*$_6^+$, Ap^R	this study
pMB114	pHT123, ΔKn^R, Tet^R	this study
pQE60	6His-tagging overexpression vector	Qiagen
pTAC3575	*phoA*$^+$, Ap^R	(Atlung *et al.*, 1991)
pUC19	cloning vector, Ap^R	(Yanisch-Perron *et al.*, 1985)
pZS-21	pSC101, $P_{LtetO-1}$, MCS-1, Kn^R	(Lutz and Bujard, 1997)
pZS*24	pSC101*, $P_{LtetO-1}$, MCS-1, Kn^R	(Lutz and Bujard, 1997)

[a] the number indicates the codon of *rovA*, in which the resistance cassette has been inserted
[b] the number indicates the codon of the corresponding gene fused to *lacZ*
[c] the number indicates the codon of the corresponding gene fused to *phoA*
