# Supplementary material for: Intrinsic Thermal Sensing Controls Proteolysis of Yersinia Virulence Regulator RovA
Source: PLoS Pathog. 2009 May 15;5(5):e1000435. doi: 10.1371/journal.ppat.1000435 (PMC2676509; doi:10.1371/journal.ppat.1000435)
Supplement: Table S2 — Oligonucleotides used in this study. (0.06 MB PDF) [file ppat.1000435.s010.pdf]

Table S2. Oligonucleotides used in this study.

Number	Sequence	Site[a]
1	GCGCGGATCCGAATCGACATTAGGATCTGATC	*Bam*HI
2	GCGGCGCTGCAGGGCTTGTTTTGTTCGCTGTG	*Pst*I
3	GCGGCGGGTACCGACTGGACTCTGGGAGTTATGC	*Kpn*I
4	CGCGATCGATGGCTTGTTTTGTTCTGCTGTG	*Cla*I
5	GCGGCGGAGCTCAATGTATTTGTACATGGAGAAAAT	*Sac*I
6	GTCCCCCGGGGAAAATTCACTGCCGGGC	*Sma*I
7	GCATACGAATTCGGTGAATAATTAACCAGATTGTCC	*Eco*RI
8	GCATACAAGCTTCGGAACGCTCAGGGTTCATAGAG	*Hind*III
9	GAGGAGCAATTGCACCATCACCATCACCATGAATCGACATTA GG	
10	CCTAATGTCGATTCATGGTGATGGTGATGGTGCAATTGCTCCTC	
11	GGTTAGAACCTTGGATCAACTGAAGGAAAAAGGTTTAATCACACGG	
12	CCGTGTGATTAAACCTTTTTCCTTCAGTTGATCCAAGGTTCTAACC	
13	GCCGCGGTCGACTGCCGCCTTCCTGCAACTCG	*Sal*I
14	GCGCGCGTCGACGCCGATGGTCAATTAATGCGCG	*Sal*I
15	GGGGGCGTCGACTCACGCCATCAACCTGTTCG	*Sal*I
16	GTGGCTGTCGACGTTCGATTATCGGTGAAGACTG	*Sal*I
17	GCGATCGTCGACTCCCCCCAAGTATTTCTTTGCG	*Sal*I
18	CGCATCGTCGACACGGTTTCAGCCGATGGTCAATTAATG	*Sal*I
19	CGCATCGTCGACAACGATTAATGTTATACAAAGTAACCC	*Sal*I
20	CGCATCGTCGACAACCTTTTTCCTCCAGTTGATCCAAG	*Sal*I
21	CGCATCGTCGACAAGACTGTTCCGTTAATTTAATTCAC	*Sal*I
22	GCGCGCGGTACCAAAGTGAAGAATACACTATGACAAATGC	*Kpn*I
23	GCGCGCATCGATAAACAGATTACTTAATCTTCATCACC	*Cla*I
24	GCCGCGTCTAGATGCCGCCTTCCTGCAACTCG	*Xba*I
25	GCGGCGCTGCAGCGCGCCAAACGCGAACTAATCG	*Pst*I
26	GCGGGGGAGCTCAACCCTGAGCGTTCCGAA	*Sac*I
27	GGGCGGCTGCAGCTATTTTGGCGCTACTGGCTG	*Pst*I
28	GCGGGGCCATGGATGAACCCTGAGCGTTCCGAAC	*Nco*I
29	GGGGCGAGATCTTTTTGGCGCTACTGGCTG	*Bgl*II
30	CCGCGCCTCGAGAATTCTCATGTTTGACAGCTTAT	*Xho*I
31	CGGCGCGAGCTCAGAATTGATTGGCTCCAATTCT	*Sac*I
32	GGTGATTTTGAACTTTTGCTTTG (Kan1)	
33	CCAGTGTTACAACCAATTAACC (Kan2)	
34	GGGCCTCGTGATACGCC (Amp1)	
35	GAGTAAACTTGGTCTGACAG (Amp2)	
36	CACAGAGGTAGACGTATC	
37	GATTTATTGTAACTAAGCAGGTG	
38	GATATTCTCACCTGCTTAG	
39	GAAAACCATCATAATTTAAATG	
40	GAAATAAAAACCGCAGCAGAG	
41	GCATTTGAAATCACCTTGACC	
42	GCCGCGTCTAGATGCCGCCTTCCTGCAACTCG	
43	GCCGCGGGATCCATTAACCTAATACGAGTATCC	
44	GCGGCGGGATCCGCGCCAAACGCGAACTAATCG	
45	GTGCTAACGACAATGAC	
46	GAGTCCAGTCTATTTTGGG	
47	GGCTACGAAATGAGCATCGC	
48	ACGATTTGTACTCCAGCGCC	

[a] The corresponding restriction sites are underlined.
