# Supplementary material for: Intrinsic Thermal Sensing Controls Proteolysis of Yersinia Virulence Regulator RovA
Source: PLoS Pathog. 2009 May 15;5(5):e1000435. doi: 10.1371/journal.ppat.1000435 (PMC2676509; doi:10.1371/journal.ppat.1000435)
Supplement: Table S3 — Primers used for the generation of deletion mutants. (0.05 MB PDF) [file ppat.1000435.s011.pdf]

Table S3. Primers used for the generation of deletion mutants.

Mutant name[a]	Primer name	Primer sequence
YP63<br>YP68	*clpP*::Kan for	GCA AAG CAA AAG TTC AAA ATC ACC CCG TCT CCT AAT AGA ATT TAC TTT GGC GCC ATT CAT AAC CGA TTC TAC TTG
	clpP::Kan rev	GGT TAA TTG GTT GTA ACA CTG GGA CTT ATT TCT GCG AGC CGA TAT ACT ATA GTG TAT GTA AAT AGA AC
	clpP for	CTT TCA GTG AGC TGA TGA ATC
	clpP rev	GAC GCT TGT AGT GGT TAT AC
YP67	*lon*::Amp for	CAC GAG ATT TCC AGT AAT CTG GCG GAA GCT AAA CTA AGA GAG AGC TCG GGC CTC GTG ATA CGC C
	lon::Amp rev	GGC GCT ACT GGC TGG GCA CCA AAG GCC GGG TGT TCC AAC GCA ATG GCG AGT AAA CTT GGT CTG ACA G
	lon for	CCT CGC ATT GTT ATC GTT G
	lon rev	CTT CTT GCG ATC CGT CAC
KB2	*hns* for	GCT CTA TTA TTA CCT CAA CAA ACC ACC CCA ATA TAA GTT TGA GAT TAC TAC GTG TAG GCT GGA GCT GCT TC
	hns rev	CAA TAA AAA ATC CCG CCG CTG GCG GGA TTT TAA GCA AGT GCA ATC TAC AAA AGC ATA TGA ATA TCC TCC TTA GT

[a] The *Y. pseudotuberculosis* mutants were constructed by adding a kanamycin resistance cassette (Kan) or an ampicillin (Amp) resistance cassette. Underlined bases correspond to the homologous nucleotides of the resistance gene (Kan or Amp). Rev: reverse primer; for: forward primer.
